# HIV-2 evades restriction by ZAP through adaptations in the U3 LTR region despite increased CpG levels

**DOI:** 10.1093/nar/gkaf826

**Published:** 2025-08-30

**Authors:** Dorota Kmiec, Rayhane Nchioua, Alexander Gabel, Asimenia Vlachou, Sabina Ganskih, Sümeyye Erdemci-Evin, Stacey A. Lapp, Diane G. Carnathan, Steven E. Bosinger, Ben Berkhout, Atze T. Das, Mathias Munschauer, Frank Kirchhoff

**Affiliations:** Institute of Molecular Virology, Ulm University Medical Center, Ulm 89081, Germany; Institute of Molecular Virology, Ulm University Medical Center, Ulm 89081, Germany; Helmholtz Institute for RNA-Based Infection Research, Würzburg 97080, Germany; Institute of Medical Virology, University Hospital Frankfurt, Goethe University, 60590 Frankfurt am Main, Germany; Institute of Molecular Virology, Ulm University Medical Center, Ulm 89081, Germany; Helmholtz Institute for RNA-Based Infection Research, Würzburg 97080, Germany; Institute of Molecular Virology, Ulm University Medical Center, Ulm 89081, Germany; Department of Pathology and Laboratory Medicine, School of Medicine, Emory University Atlanta, GA 30322, United States; Department of Pathology and Laboratory Medicine, School of Medicine, Emory University Atlanta, GA 30322, United States; Department of Pathology and Laboratory Medicine, School of Medicine, Emory University Atlanta, GA 30322, United States; Amsterdam University Medical Center, University of Amsterdam, Medical Microbiology and Infection Prevention, 1105AZ, Amsterdam, Netherlands; Amsterdam University Medical Center, University of Amsterdam, Medical Microbiology and Infection Prevention, 1105AZ, Amsterdam, Netherlands; Helmholtz Institute for RNA-Based Infection Research, Würzburg 97080, Germany; Institute of Medical Virology, University Hospital Frankfurt, Goethe University, 60590 Frankfurt am Main, Germany; Heidelberg University, Medical Faculty Heidelberg, Department of Infectious Diseases, Molecular Virology, Center for Integrative Infectious Disease Research, D-69120 Heidelberg, Germany; Institute of Molecular Virology, Ulm University Medical Center, Ulm 89081, Germany

## Abstract

Simian immunodeficiency viruses infecting sooty mangabeys (SIVsmm) gave rise to nine groups of human immunodeficiency virus type 2 (HIV-2). Two of these (A and B) spread substantially with an estimated 1–2 million individuals affected. The evolutionary adaptations that facilitated HIV-2’s spread in humans are still poorly understood. Here, we report that diverse SIVsmm strains efficiently infect primary human T cells. However, they are more sensitive to interferon than HIV-2, indicating that interferon-stimulated genes (ISGs) pose a barrier to the successful spread of SIVsmm in humans. One of the best-known antiviral ISGs is the zinc finger antiviral protein (ZAP), which targets CpG dinucleotides in RNA. To evade ZAP-mediated restriction, many viruses, including HIV-1, suppress their CpG content. Unexpectedly, we found that HIV-2 is more resistant to ZAP restriction than HIV-1 and SIVsmm despite having 33% more CpGs. Identification of ZAP-binding sites using RNA enhanced crosslinking immunoprecipitation and analyses of chimeric HIV-2/SIVsmm viruses revealed that the determinants of ZAP resistance map to the *nef*/U3 region and promote HIV-2 replication in primary human T cells. Our results indicate that HIV-2 evolved a CpG-independent ZAP resistance mechanism which might have been facilitated by relaxed functional constraints acting on Nef in the human host.

## Graphical Abstract

**Graphical Abstract:**
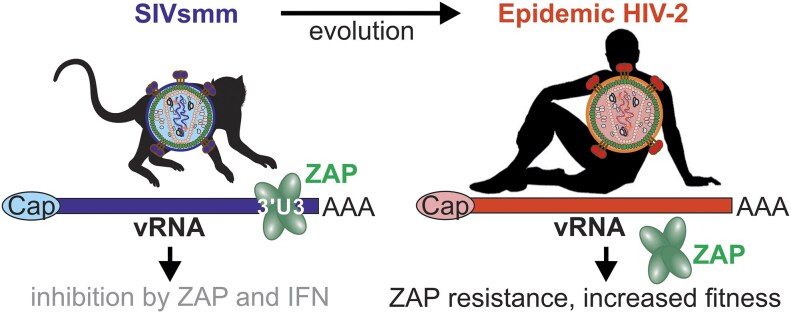

## Introduction

Zinc finger antiviral protein (ZAP), also known as ARTD13, PARP13, and ZC3HAV1, is a broadly expressed cellular RNA-binding protein that inhibits multiple RNA and DNA viruses [1–3]. Like other antiviral restriction factors that constitute intrinsic immunity, ZAP evolved under positive selection and its expression is upregulated by interferons (IFNs) [3–6]. ZAP binds to CpG dinucleotides in HIV-1 RNAs and recruits the co-factors ubiquitin ligase tripartite motif containing 25 (TRIM25) and endoribonuclease KH and NYN domain containing (KHNYN) to degrade it, which in turn reduces viral protein expression and replication [7–10]. CpGs are the least abundant dinucleotide type found in the transcriptomes of most vertebrates, including humans [2, 11, 12]. ZAP exploits this CpG suppression to distinguish between self and non-self RNAs and target viral transcripts.

The continuous arms race between viruses and their hosts shapes pathogen evolution and enables the evasion of antiviral restriction factors such as ZAP. Many successful viruses mimic the CpG suppression of their host’s genomes to evade the recognition by ZAP. For example, HIV-1 suppresses its genomic CpG content by over 80%, which is even lower than CpG suppression of the human transcriptome (60%) [7, 13]. However, the CpG content of primate lentiviral genomes varies greatly, and does not correlate with ZAP sensitivity [13], suggesting alternative ZAP evasion mechanisms.

Simian immunodeficiency viruses (SIVs) naturally infect over 40 African primate species [14]. Of those, SIVs infecting chimpanzees, gorillas, and sooty mangabeys successfully crossed the interspecies barrier and gave rise to HIV-1 (SIVcpz and SIVgor) and HIV-2 (SIVsmm). While the HIV-1 group M originating from SIVcpz has spread globally causing >95% of all HIV infections, HIV-2 infects ∼1–2 million people, mostly in West Africa [15]. HIV-1 and HIV-2 evolved sophisticated ways to evade or counteract human immune responses and cause persistent infection that, if untreated, eventually leads to a fatal acquired immune deficiency syndrome (AIDS). SIVsmm, the ancestor of HIV-2, does not cause disease in its natural host and is highly prevalent in sooty mangabeys, which are hunted for bushmeat or kept as pets in parts of West Africa [16]. SIVsmm strains infect human T cells *in vitro* and crossed the species barrier from monkeys to humans on at least nine occasions [17–19]. However, only two of these zoonotic transmissions spread among humans, giving rise to epidemic HIV-2 groups A and B. While the overall prevalence of HIV-2 is declining, new infections with HIV-2 groups A, B, and their recombined form (AB) continue to be reported outside of West Africa [20–22]. HIV-2 and SIVsmm share the same genomic organization but recent findings suggest that their immunomodulatory functions, especially those performed by accessory proteins, differ [17, 19, 23]. The specific evolutionary changes acquired by the epidemic groups of HIV-2 during their adaptation to humans remain to be defined.

Here, we examined whether ZAP poses a barrier to effective spread of SIVsmm in human cells. We found that SIVsmm strains are more sensitive to IFN than HIV-2 and also significantly more susceptible to inhibition by ZAP. Surprisingly, epidemic HIV-2 evolved resistance to ZAP despite acquiring additional CpG dinucleotides during its adaptation to humans. Through enhanced crosslinking immunoprecipitation (eCLIP) analysis of ZAP-binding sites (ZBSs) in viral RNA and exchanging specific genomic regions between HIV-2 and SIVsmm, we show that HIV-2’s evasion of ZAP is not determined by the *env* gene coding region, as observed in HIV-1 [13]. Instead, it involves changes in the U3 region of the LTR that overlaps with the *nef* gene. This region encodes the C-loop of Nef, which includes an ExxxLL motif. In SIVsmm, this part of Nef is critical for CD4 down-modulation [24], as well as antagonism of SERINC5 [19] and tetherin [17, 25]. While the ExxxLL motif and CD4 downmodulation are conserved in HIV-2 Nefs, natural alterations in this region impair antagonism of SERINC5 and tetherin [17, 19]. Instead, HIV-2 uses Env to counteract these host restriction factors [17, 26]. Our results suggest that reduced functional constraints acting on HIV-2 Nef facilitated adaptive CpG-independent changes in the overlapping U3 region of the LTR that confer ZAP resistance. These changes enhance HIV-2 replication in human cells and may have contributed to its epidemic spread.

## Materials and methods

### Cell lines

HEK293T and HeLa cells were obtained from ATCC. HEK293T ZAP KO cells have been previously described [7]. Jurkat WT and ZAP KO cells were described before [27, 28]. TZM-bl cells express CD4, CCR5, and CXCR4 and contain the β-galactosidase genes under the control of the HIV promoter [29, 30].

Adherent cell lines were cultured in Dulbecco’s modified Eagle’s medium (DMEM) supplemented with 2.5% (during virus production) or 10% (at all other times) heat-inactivated fetal calf serum (FCS), 2 mM L-glutamine, 100 U/ml penicillin, and 100 μg/ml streptomycin. Jurkat cells were cultured in RPMI supplemented with 10% FCS, 2 mM L-glutamine, 100 U/ml penicillin, and 100 μg/ml streptomycin.

### Isolation of primary cells

Peripheral blood mononuclear cells (PBMCs) or CD4 + T cells from healthy human donors were isolated using lymphocyte separation medium (Biocoll separating solution; Biochrom) or RosetteSep Human CD4 + T Cell Enrichment Cocktail (Stemcell), stimulated for 3 days with phytohemagglutinin (PHA, 2 μg/ml), and 10 ng/ml interleukin-2 (IL-2) in RPMI 1640 medium containing 10% FCS. Where indicated, cells were stimulated with IFNα 24 h prior to infection.

### Expression constructs

The pCG IRES eBFP vector encoding human ZAP-L containing an N-terminal hemagglutinin (HA) tag and empty vector control were described before [13]. The infectious molecular clones (IMCs) of HIV-1 NL4-3, HIV-1 NL4-3 CpG high mutant, CH77TF, CH58 TF, THRO, CH167, HIV-2 7312A, ST, KR, GH123, ROD10, MCR, as well as SIVsmm PGM, SIVsmm PGM nef-IRES eGFP, L1.V1, L1.V2, L2, L3, L4, and L5 were described previously [8, 23, 31–43]. HA-ZAP-L zinc-finger 1–4 deletion mutant was generated by Q5 site-directed mutagenesis using pCG HA-ZAP-L IRES BFP as a template. The pCG mCherry-ZAP-L IRES BFP expression construct was generated by Gibson assembly using the XbaI/Mlul cloning sites. SIVsmm Env and Nef expression vectors and HIV-2 7312A *env* chimeras were previously reported [17]. HIV-2 7312A IRES eGFP constructs carrying premature stop codons in *vpr* and *nef* ORFs were described before [35]. SIVsmm Vpr and Nef expression constructs were described before [19, 44]. Chimeric HIV-2 and SIVsmm IMCs as well as CpG mutants thereof were generated using Splicing by Overlap Extension PCR (SOE PCR) or Gibson assembly (NEB) and cloned into pCR XL TOPO or pBR vector using NotI/MluI restriction sites. All constructs were cloned using primers (from biomers.net) listed in Table 1 and their correctness was confirmed by Sanger sequencing (eurofins).

**Table 1.** Oligonucleotides used for cloning

Construct	Primers used	Cloning type
pCG HA-ZAP-L ZnF1-4 deletion IRES BFP	fwd aacctgatggacagaaaggtgc rev gacccgggctcgagtggtgg	SDM
pCG mCherry-ZAP-L IRES BFP	fwd 1 tgtagaagcgcgtaggccttctagatatggtgagcaagggcgagga rev 1 ccgggtccgccttgtacagctcgtccatgcc fwd 2 gctgtacaaggcggacccggaggtgtgctg rev 2 tgtagtactccgggatccgactaactaatcacgcaggctttgtcttcagtatattcaatcacatattgtg	Gibson assembly
pCG sooty mangabey ZAP-L IRES BFP	fwd 1 gccgcggcagagccagcacagccagc rev 1 gaatggttatggcatccttctgacgc fwd 2 tgtagaagcgcgtaggccttctagatatgtacccatacgatgttccagattacgctgcggacccggaggtg rev 2 gtagtactccgggatccgacgcgtctaactaatcacgcaggg	Blunt TOPO cloning and Gibson assembly
5′HIV-2 7312A/3′ SIVsmmL1.V1	fwd 1 acgccaagctatttaggtgacgcgtggaagggattttttatagtgaaagaagacataggatattagatacatattttgag fwd 2 aggcctagcctaaaatgacagaaagacctcc rev 1 tgtcattttaggctaggcctgggggagg rev 2 ctctagatgcatgctcgagcggccgctgctagggattttcctgc	Gibson assembly
5′SIVsmmL1.V1/ 3′HIV-2 7312A	fwd 1 acgccaagctatttaggtgacgcgtggaagggatttattacag fwd 2 aggactagcataatggcagaagcagcctc rev 1 tctgccattatgctagtcctggaggggg rev 2 ctctagatgcatgctcgagcggccgctgctagggattttcctgc	Gibson assembly
HIV-2 7312A + SIVsmm L1.V1 Nef	fwd 1 aagataataggactattataagcttgaataagtattataatttaacaatacattgtaag fwd 2 tacctccaatatgggtggcattacctcc fwd 3 aacaagctgaacaggaaagcagcagcataaag rev 1 tgccacccatattggaggtacgcaaacag rev 2 gctttcctgttcagcttgtttccttcttgttag rev 3 ctctagatgcatgctcgagcccgctgctagggattttc	Gibson assembly
HIV-2 7312A + SIVsmmL2 Nef	fwd 1 aagataataggactattataagcttgaataagtattataatttaacaatacattgtaag fwd 2 tacctccaatatgggtgccagtggctcc fwd 3 aacaagctgaacaggaaagcagcagcataaag rev 1 tggcacccatattggaggtacgcaaacag rev 2 gctttcctgttcagcttgtttccttcttgtcagc rev 3 ctctagatgcatgctcgagcccgctgctagggattttc	Gibson assembly
HIV-2 7312A + SIVsmmL1.V1 LTRs and SIVsmmL1.V1 + HIV-2 7312A LTRs	fwd 1 gccaacctgctagggattttcctgc rev 1 gcaggttggcgcccgaacagggacccgag fwd 2 gactggaagggatttattacagtg rev 2 ccagtccccccttttcttttataaaatgagac fwd 3 gcaggttggcgcccgaacagggac rev 3 caacctgctagggattttcctgc	SOE PCR
HIV-2 7312A + SIVsmm L1.V1 3′ U3	fwd 1 gacttagaagaggctatagaccggttttctcttccccccc fwd 2 aaggggggactggaagggatttattacagtg fwd 3 gctctgtattcagtcgctctgcggagaggc rev 1 atcccttccagtccccccttttcttttataaaatgagacatg rev 2 agagcgactgaatacagagcgaaatgcagttg rev 3 ctctagatgcatgctcgagcgctgctagggattttcctgcttagg	Gibson assembly
HIV-2 7312A + SIVsmm L1.V1 3′ R	fwd 1 gacttagaagaggctatagaccggttttctcttccccccc fwd 2 gcattgtattcagtcactctgcggagaggc fwd 3 tttagaagtaagtcaagtgtgtgttcccatc rev 1 agagtgactgaatacaatgcaagaagcgggtacatttatac rev 2 acacttgacttacttctaaatggcagctttattgaagagg rev 3 ctctagatgcatgctcgagcgctgctagggattttcctgc	Gibson assembly
HIV-2 7312A + SIVsmm L1.V1 3′ U5	fwd 1 gacttagaagaggctatagaccggttttctcttccccccc fwd 2 attagaagcaagctagtgtgtgttcccatc rev 1 cacactagcttgcttctaattggcagctttattaagagg rev 2 ctctagatgcatgctcgagcgctgctagggattttcctgc	Gibson assembly
HIV-2 7312A + SIVsmmL1.V1 U3 fragment A	fwd 1 gacttagaagaggctatagaccggttttctcttccccc rev 1 tcactgtaataaatcccttccagtcccc fwd 2 gaagggatttattacagtgaaaggagacataaaatattag rev 2 actcttctgggtacttaacaaatgccttg fwd 3 tgttaagtacccagaagagtttgggtatcagtcaggattac rev 3 ctctagatgcatgctcgagcgcggccgctgctagggat	Gibson assembly
HIV-2 7312A + SIVsmmL1.V1 U3 fragment B	fwd 1 gacttagaagaggctatagaccggttttctcttccccc rev 1 tgacatactaccaaactcttctgggaacc fwd 2 aagagtttggtagtatgtcaggattgcc rev 2 cagtgtcagcttgtttccttcttgttagc fwd 3 aaggaaacaagctgacactgcagggactttcc rev 3 ctctagatgcatgctcgagcgcggccgctgctagggat	Gibson assembly
HIV-2 7312A + SIVsmmL1.V1 U3 fragment C	fwd 1 gacttagaagaggctatagaccggttttctcttccccc rev 1 ccctgctgtctcagccagtttcctttcttatg fwd 2 aactggctgagacagcagggactttccac rev 2 actgaatacagagcgaaatgcagttgtatttatac fwd 3 catttcgctctgtattcagtcgctctgcggagag rev 3 ctctagatgcatgctcgagcgcggccgctgctagggat	Gibson assembly
SIVsmmL1.V1 + HIV-2 7312A U3 region A	fwd 1 cagtgagaccaagagtccccttaagaaccatgacatacaaattgg rev 1 tcactataaaaaatcccttccagtcccc fwd 2 gaagggattttttatagtgaaagaagacataggatattagatac rev 2 ctcttctgggaacctgttgaaggcctcatc fwd 3 tcaacaggttcccagaagagtttggtag fwd 3 gcttaattaagtttaaacgctgctagggattttcctgc	Gibson assembly
SIVsmmL1.V1 + HIV-2 7312A U3 region B	fwd 1 cagtgagaccaagagtccccttaagaaccatgacatacaaattg rev 1 tgactgatacccaaactcttctgggtac fwd 2 aagagtttgggtatcagtcaggattacc rev 2 ctgtctcagccagtttcctttcttatgcag fwd 3 aaggaaactggctgagacagcagggactttcc rev 3 gcttaattaagtttaaacgctgctagggattttcctgc	Gibson assembly
SIVsmmL1.V1 + HIV-2 7312A U3 region C	fwd 1 cagtgagaccaagagtccccttaagaaccatgacatacaaattg rev 1 ccctgcagtgtcagcttgtttccttcttg fwd 2 aacaagctgacactgcagggactttccag rev 2 actgaatacaatgcaagaagcgggtacatttatac fwd 3 cttcttgcattgtattcagtcactctgc rev 3 gcttaattaagtttaaacgctgctagggattttcctgc	Gibson assembly
HIV-2 7312A + SIVsmm CLIP peak 1	Fwd gacttagaagaggctatagaccggttttctcttccccccc Rev ctctagatgcatgctcgagcggccgctgctagggattttcctgc	Gibson assembly
HIV-2 7312A + SIVsmm CLIP peak 2	Fwd 1 gacttagaagaggctatagaccggttttctcttccccc Rev 1 gcttcatctgagacatttactggcactagtttccacagccagc Fwd 2 aaatgtctcagatgaagctcaggaagatgagacacatcgcttgatgcatccagc Rev 2 ctctagatgcatgctcgagcgcggccgctgctagggat	Gibson assembly
HIV-2 7312A + U3 CpG 1	fwd 1 gacttagaagaggctatagaccggttttctcttccccc rev 1 gatgggtctcctcttcctcccgggttac fwd 2 gaagaggagacccatcgtctagtgcacccagcacag rev 2 ctctagatgcatgctcgagcgcggccgctgctagggat	Gibson assembly
SIVsmmL1.V1 - U3 CpG 1	fwd 1 cagtgagaccaagagtccccttaagaaccatgacatacaaattgg rev 1 caatgtgtctcatcttcctgagcttcatctg fwd 2 gaagatgagacacattgcttgatgcatccagcac rev 2 gcttaattaagtttaaacgctgctagggattttcctgc	Gibson assembly
HIV-2 7312A + U3 CpG 2	fwd 1 gacttagaagaggctatagaccggttttctcttccccccc rev 1 tgcctcttgcgtttagtctagccttccattccttctc fwd 2 tagactaaacgcaagaggcatacctacagag rev 2 ctctagatgcatgctcgagcggccgctgctagggattttcctgcttag	Gibson assembly
SIVsmmL1.V1 - U3 CpG 2	fwd 1 cagtgagaccaagagtccccttaagaaccatgacatacaaattg rev 1 ggcctcttgctgttagccttctctttacc fwd 2 aaggctaacagcaagaggccttttaaaaatg rev 2 gcttaattaagtttaaacgcggccgctgctagggattttcctgc	Gibson assembly
HIV-2 7312A + U3 CpG 3	fwd 1 gacttagaagaggctatagaccggttttctcttccccccc rev 1 aatacaatgcgagaagcgggtacatttatac fwd 2 cccgcttctcgcattgtattcagtcgctctg rev 2 ctctagatgcatgctcgagcggccgctgctagggattttcctgcttag	Gibson assembly
SIVsmmL1.V1 - U3 CpG 3	fwd 1 cagtgagaccaagagtccccttaagaaccatgacatacaaattg rev 1 aatacagagcaaaatgcagttgtatttatacag fwd 2 actgcattttgctctgtattcagtcactc rev 2 gcttaattaagtttaaacgcggccgctgctagggattttcctgc	Gibson assembly

### Generation of sooty mangabey ZAP

Whole blood from two sooty mangabeys was collected and PBMCs were purified via Ficoll density gradient centrifugation and RNA from 1 million cells was extracted according to the manufacturer’s instructions (RNeasy Mini Kit, Qiagen cat# 74104).

To generate complementary DNA (cDNA) from the extracted RNA , the SuperScript^™^ IV Reverse Transcriptase (cat# 18090010, Thermo Fisher Scientific) kit was used. The cDNA was cleaned using a DNA Clean and Concentrator spin column (Zymo, cat# D4033) and eluted in RNase-free water. ZAP transcript was amplified from cDNA using primer pair binding in the 5′ and 3′ ZAP-L UTR. Resulting PCR product was ligated into pCR XL TOPO Blunt vector. Sanger sequencing revealed three unique ZAP-L alleles. In the next step, ZAP with N-terminal HA tag was cloned into the pCG IRES BFP vector using XbaI/MluI restriction sites. All constructs were cloned using primers listed in Table 1 and their correctness was confirmed by Sanger sequencing (eurofins).

### Transduction of Jurkat cells

Vesicular stomatitis virus glycoprotein (VSV-g)-pseudotyped virus stocks were prepared by transfecting HEK293T cells with 1 μg of VSV-g and 5 μg proviral DNA construct, followed by a medium change. Supernatants were harvested 48 h later and normalized based on infectivity as measured by the TZM-bl reporter cells. One million Jurkat WT or ZAP KO cells were seeded and treated with IFN-α (200 U/ml) or left unstimulated at the time of transduction. The input virus was removed 24h later, and cells were washed in PBS. Three and six days later, supernatants were harvested and used to infect TZM-bl reporter cells in triplicates. β-Galactosidase activity was measured 2 days later using the Gal-Screen kit (Applied Biosystems) as relative light units per second using a microplate luminometer.

### Flow cytometry of primary cells

PHA-activated PBMCs were infected with VSV-G-pseudotyped HIV-2 or SIVsmm viruses and harvested three days post-infection for flow cytometric analysis. Cells were fixed and permeabilized (NordicMUBio, Cat#GAS-002) and then stained intracellularly for Gag (FITC conjugated; 6604665; Beckman Coulter) and ZAP (GenTex, cat# GTX120134), followed by secondary anti-rabbit AlexaFluor647 (Invitrogen, cat#A-21245). Rabbit isotype IgG antibody served as a negative control (CellSignaling cat#2729S). The AF647 mean fluorescence intensity (MFI) of infected (FITC+) cells was measured using FACSCanto II cytometer and the values were used to determine changes in ZAP protein levels.

To determine the effect of IFN stimulation on ZAP, TRIM, and KHNYN expression, PBMCs were treated with 500 u/ml type I IFN (α, β) or 200 u/ml of type II IFN(γ) for 48 h. Cell were then fixed and permeabilized (NordicMUBio, Cat#GAS-002) and stained intracellularly for ZAP (GenTex, cat# GTX120134) TRIM25 (BD, cat#610570), or KHNYN (SantaCruz, cat#sc-514168) followed by secondary anti-rabbit or anti-mouse AlexaFluor647 (Invitrogen). Rabbit and mouse isotype IgG antibodies served as a negative controls. The AF647 MFI of live single cells was measured using the FACSCanto II cytometer and the values were used to determine n-fold changes in protein levels compared to the untreated condition.

### Flow cytometry of transfected HEK293T cells

To determine the effect of ZAP on the expression of HIV-2 and SIVsmm *vpr*, *ned*, and *env* ORFs, HEK293T ZAP KO cells in 24-well format were co-transfected with either 0.5 μg pCG ZAP-L IRES BFP or 0.5 μg pCG ZAP-L IRES BFP mutant lacking the four zinc fingers responsible for vRNA binding (negative control) and 0.5 μg of pCG IRES eGFP or pCAGGS IRES eGFP reporter constructs expressing viral Nef, Vpr, and Env. In these reporter constructs, the viral protein is expressed from the same mRNA as the GFP reporter protein due to the presence of an IRES element. Reporter SIVsmm IRES eGFP and HIV-2 7312A IRES eGFP proviruses were also co-transfected with pCG ZAP-L IRES BFP or 0.5 μg pCG ZAP-L delZnF4 IRES BFP mutant as additional controls. Two days after the transfection, the cells were fixed in 1% Paraformaldehyde (PFA) in Phosphate Buffered Saline (PBS) and GFP expression levels within the BFP + cell population was measured using a FACSCanto II cytometer. BFP/GFP compensation was set using single-fluorophore transfected HEK293T cells. MFI of GFP reporter in the presence of ZAP-L was normalized to the MFI of GFP reporter in the presence of ZAP-L delZnF4 for each viral ORF reporter construct to obtain the eGFP MFI [%] value.

### Metabolic activity assay

To measure the effect of ZAP KO on cell viability/metabolic activity, HEK293T WT or HEK293T ZAP KO cells were seeded at 0.35 mln/ml into 24-well plates and incubated for 3 days. For Jurkat cells, 0.3 mln WT or ZAP KO cells were seeded into wells of 96-well plate and incubated for 3 days. To measure the effect of ZAP overexpression, ZAP KO HEK293T cells were seeded at 0.35 mln/ml into 24-well plate and transfected with increasing concentrations of pcDNA3.1 or pCG IRES BFP-based ZAP expression vector up to 1μg, or control vector expressing just GFP or BFP, and incubated for 2 days. To measure the metabolic activity/viability of cells, methyl-thiazolyl blue–tetrazolium bromide salt (MTT-salt) was added to cells at 1:10 salt to medium ratio. The cells were incubated at 37°C for 3 h. Formed salt crystals were dissolved in 1 ml of dimethyl sulfoxide/ethanol solution. Absorbance was measured at 490–650 nm using a microplate reader.

### Confocal microscopy

Hela cells were seeded onto poly-Lysine coated IBIDI-slides and co-transfected with 125 ng of pCG mCherry-ZAP-L expression plasmid and 125 ng of HIV-2 7312A IRES eGFP provirus or empty vector control LT1 transfection reagent. Forty hours post-transfection, cells were fixed in PBS containing 2% PFA and 0.05% TritonX and stained for 30 min in 1 μg/ml 4′,6-diamidino-2-phenylindole (DAPI).

### qRT PCR

To determine the impact of ZAP on viral transcript levels, RNA from ZAP and provirus co-transfected HEK293T ZAP KO cells was isolated using RNeasy Plus Mini Kit (Cat#74134), according to the manufacturer’s instructions. RNA concentration was measured using Nanodrop and normalized. Real-time quantitative PCR (RT-qPCR) was performed using TaqMan Fast Virus 1-step master mix (Thermo Fisher, cat# 4444436). The sequences of the primers and probe for HIV-2/SIVsmm *nef*/U3 region were as follows: forward primer: 5′-tgacatacaaattggcaatagacat-3′; reverse primer, 5′-atctaatatyytatgycttctttcac-3′; probe, 5′-FAM (6-carboxyfluorescein)-agaaaaggggggactggaagggatt-TAMRA (6-carboxytetramethylrhodamine)-3′. Primers were purchased from Biomers (Ulm, Germany). Proviral DNA constructs of HIV-2 and SIVsmm were used as a quantitative standard to obtain viral copy numbers. All reactions were run in technical duplicates.

### Lentiviral sensitivity to ZAP

To assess relative lentiviral sensitivity to ZAP overexpression, HEK293T ZAP KO cells (in 24-well format) were co-transfected using polyethyleneimine Max (PEI Max, Polysciences) transfection reagent with 600 ng of the indicated provirus and increasing concentrations (0, 100, 200, and 400 ng) of pCG HA-ZAP-L IRES BFP expression vector and the amount of DNA was normalized to 1 μg by adding pCG IRES GFP vector. Virus-containing supernatants were harvested 2 days later and used to infect 10 000 TZM-bl reporter cells in triplicates. β-Galactosidase activity was measured 2–3 days later using the Gal-Screen kit (Applied Biosystems) as relative light units per second using a microplate luminometer. Infectious virus yield values of each virus in the presence of ZAP were normalized to the corresponding GFP control.

### Lentiviral sequence analysis, CpG mapping, and RNA structure prediction

Sequences of SIVsmm and HIV-2 isolates were obtained from the Los Alamos HIV sequence database (https://www.hiv.lanl.gov/content/sequence/HIV/mainpage.html). CpG frequency was calculated as [number of CpG]/[sequence length], whereas CpG suppression was calculated as [number of CpG * sequence length]/[number of C * number of G]. The RNAfold web server based on the latest ViennaRNA Package (Version 2.6.3; http://rna.tbi.univie.ac.at/cgi-bin/RNAWebSuite/RNAfold.cgi) was used to predict the minimum free energy (MFE) RNA structure, using the dynamic programming algorithm [45].

### Western blotting

To examine the viral protein levels under the expression of ZAP, HEK293T ZAP KO cells were co-transfected in 12-well plates with 1.25 μg of indicated provirus and 0.25 μg of pCG HA-ZAP-L IRES BFP vector or empty pCG IRES BFP vector. Two days post-transfection, cells were lysed with coimmunoprecipitation (CO-IP) buffer (150 mM NaCl, 50 mM HEPES, 5 mM EDTA, 0.10% NP-40, 0.5 mM sodium orthovanadate, 0.5 mM NaF, protease inhibitor cocktail from Roche), and cell-free virions were purified by centrifugation of cell culture supernatants through a 20% sucrose cushion at 20 800 × *g* for 90 min at 4°C and lysed in CO-IP lysis buffer. Samples were reduced in the presence of β-mercaptoethanol by boiling at 95°C for 10 min. Proteins were separated in 4% to 12% Bis-Tris gradient acrylamide gels (Invitrogen), blotted onto a polyvinylidene difluoride (PVDF) membrane, and incubated with anti-HIV-1 Env (cat#ADP20421, CFAR), anti-HIV-2 Env (cat#ARP-1410; NIH AIDS Reagent Program), anti-HIV-1 p24 (cat#ab9071; Abcam), anti-SIVmac p27 (cat#ARP-2321; NIH AIDS Reagent Program), anti-GAPDH (Cat# 607 902; BioLegend), anti-ZAP (cat#. GTX120134; GeneTex), anti-KHNYN (#sc-514168, Santa Cruz Biotechnology), anti-TRIM25 (cat#610570, BD), and anti-Hsp90 (cat#sc7947, Santa Cruz Biotechnology) antibodies. Subsequently, blots were probed with IRDye 680RD goat anti-rabbit IgG(H + L) (cat# 926-68071; LI-COR) and IRDye 800CW goat anti-mouse IgG(H + L) (cat# 926-32210; LI-COR) Odyssey antibodies and scanned using a Li-Cor Odyssey reader.

### LTR activity reporter assay

To determine the effect of ZAP on the activity of HIV-2 and SIVsmm LTR promoters, 20 000 ZAP KO HEK293T cells/well were seeded in 96-well plates and co-transfected in triplicates with firefly luciferase reporter constructs (30 ng) under the control of the HIV or SIVsmm LTR and expression constructs for ZAP (60 ng) or a control vector (60 ng). At 40 h post-transfection, cells were lysed and firefly luciferase activity was determined using the Luciferase Assay Kit (Promega) according to the manufacturer’s instructions and Orion microplate luminometer (Berthold).

### Enhanced crosslinking and immunoprecipitation sequencing

HEK293T ZAP KO cells (8 mln) were co-transfected with 30 μg of pCG HA-ZAP-L IRES BFP and 50 μg of HIV-2 7312A or SIVsmmL1.V1 proviral DNA. After 6h, medium was changed and cells were incubated at 37°C 5% CO_2_ for another 40 h. The culture medium was removed, and cells were washed once with ice-cold PBS followed by crosslinking (254 nm and 0.8 J/cm^2^ UV light) on ice. Cells were harvested into ice-cold PBS and centrifuged at 400 × *g* at 4°C for 5 min. The cell pellet was washed once with ice-cold PBS and frozen down at −80°C. Frozen cell pellets were lysed in 50 mM Tris–HCl pH 7.5, 150 mM NaCl, 1 mM EDTA, 1% (vol/vol) NP-40, 0.5% sodium deoxycholate, 0.25 mM TCEP [Tris(2-carboxyethyl) phosphine hydrochloride], and complete EDTA-free protease inhibitor cocktail (Roche).

Immunoprecipitation and cDNA library preparation was performed as described in the eCLIP method [46], including the following modifications: immunoprecipitates were washed two times in 1 ml CLIP lysis buffer and two times in IP wash buffer [50 mM Tris–HCl pH 7.4, 300 mM NaCl, 1 mM EDTA, 1% (v/v) NP-40, 0.5% sodium deoxycholate, and 0.25 mM TCEP], followed by two washes in 50 mM Tris–HCl pH 7.4, 1 mM EDTA, and 0.5% (v/v) NP-40. All other steps were carried out as described in the eCLIP method. Briefly, following cell lysis, unprotected RNA was digested with RNase I, and HA-tagged ZAP proteins were immunoprecipitated using a mouse monoclonal HA-antibody (Abcam, ab49969). Following immunoprecipitation, protein–RNA complexes were separated by SDS–PAGE and transferred onto a nitrocellulose membrane. Bands migrating at the expected size range were excised for immunoprecipitation (IP) and size-matched input (SMI) samples. Crosslinked RNA fragments were released by Proteinase K digestion and recovered RNA fragments were converted into a cDNA library for both IP and SMI samples by following the eCLIP procedure (PMID: 27018577). Sequencing was performed using the Illumina NextSeq 500 platform.

### eCLIP data analysis

Resulting paired-end sequencing libraries with read lengths of 2 × 40 nucleotides were adapter- and quality trimmed using cutadapt (v1.18). Reads <18 nt were discarded. A custom java program was applied to identify and clip the unique molecular identifier (UMI) associated with each read. The trimmed reads were then aligned to the human (hg38, Ensembl release 110) and one of the respective viral genomes (gRNA sequence based on published full genome sequences SIVsmm: GenBank KU182922.1; HIV-2: GenBank KX174311.1) using STAR (v2.7.10a) [47] with the parameters –outFilterScoreMinOverLread 0 –outFilterMatchNminOverLread 0 –outFilterMatchNmin 0 –outFilterType Normal –alignSoftClipAtReferenceEnds No –alignSJoverhangMin 8 –alignSJDBoverhangMin 1 –outFilterMismatchNoverLmax 0.04 –scoreDelOpen − 1 –alignIntronMin 20 –alignIntronMax 3000 –alignMatesGapMax 3000 –alignEndsType EndToEnd. PCR duplicates were removed with the UMI-aware deduplication functionality in Picard's MarkDuplicates. Protein binding regions were predicted by using MACS2 [48], which models read coverage enrichment in an IP sample over a paired SMI control under a Poisson distribution. The analysis was performed using the parameters: -s 31 –keep-dup all –nomodel –d-min 25 –call-summits –scale-to small –shift 25 –nolambda –extsize 5 –max-gap 20 –min-length 5. The identified MACS2 peaks were further filtered by applying a one-sided Fisher’s exact test. Statistically significant enrichment was determined by calculating the odds ratio of mapped reads within each peak against all remaining mapped reads between IP and SMI. Multiple testing correction was applied using the Benjamini–Yekutieli [49] procedure, and only peaks with an adjusted *P-*value < 0.05 and a log2 foldchange > 0.5 were considered for further analysis. Additionally, the enrichment of regions of interest, such as R, U5, and U3, was calculated by using a one-sided Fisher’s exact test, following the same approach as the filtering of MACS2 peaks.

To visualize the eCLIP signal in IP relative to SMI, the relative information content of IP over SMI [50–52] was calculated as *p_i_* × log_2_(*p*_i_*/q*_i_), where *i* represents a genomic position, *p_i_* denotes the relative fraction of aligned reads covering that position in IP, and *q_i_* denotes the relative fraction of aligned reads covering the same position in SMI. The relative information content was visualized using the Integrative Genome Visualization (IGV) Browser.

### Nucleotide identity analysis

HIV-2 and SIVsmm curated *nef* nucleotide sequence alignment (2022 version) was downloaded from the Los Alamos HIV database (https://www.hiv.lanl.gov/content/sequence/). The reference (SIVmac) as well as duplicate and incomplete sequences were removed. Sequences were codon aligned for HIV-2 and SIVsmm set and the identity of sequence pairs within each set were calculated using “Ident and Sim” online tool [53] (https://www.bioinformatics.org/sms2/ident_sim.html).

### Statistical analysis

Statistical analysis was performed using GraphPad Prism software. ANOVA, Mann–Whitney *U*-test or two-tailed Student’s *t*-test were used to determine statistical significance. Significant differences are indicated as: *, *P* < 0.05; **, *P* < 0.01; ***, *P* < 0.001. Statistical parameters are specified in the figure legends.

## Results

### SIVsmm is highly sensitive to restriction by type I IFN in primary human cells

**Figure 1.**
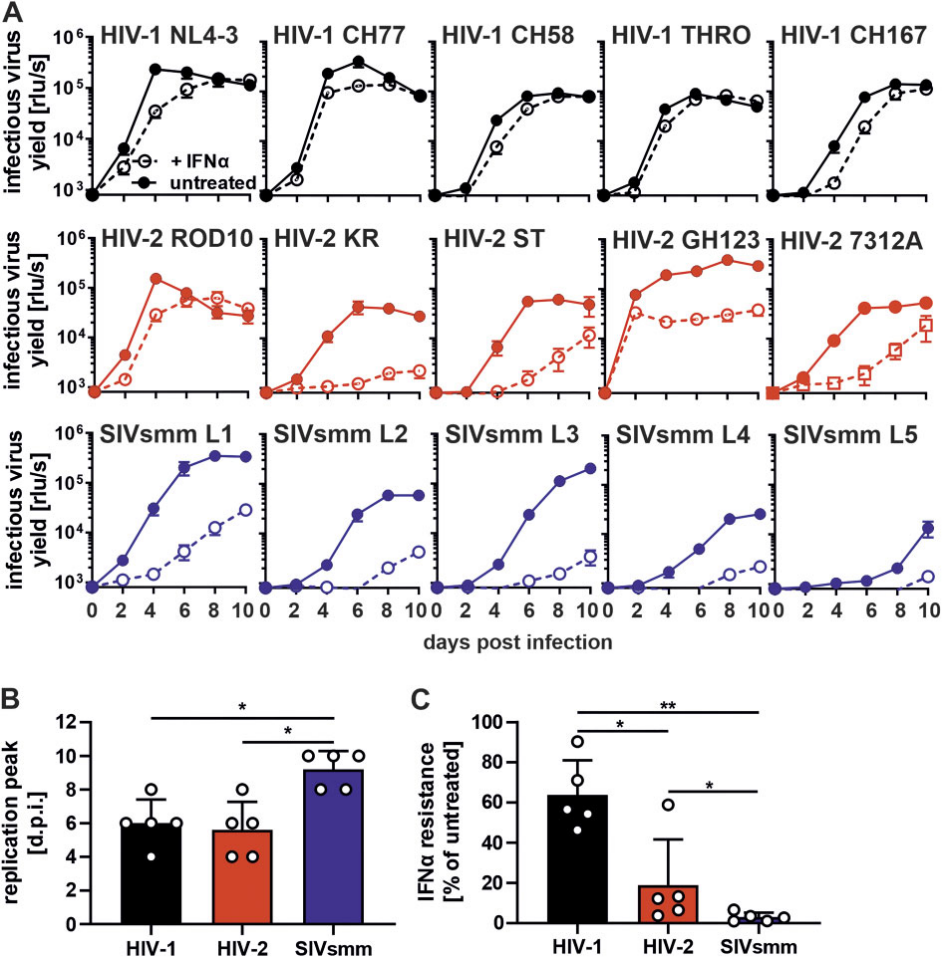
Replication fitness and type I IFN sensitivity of HIV-1, HIV-2, and SIVsmm in primary human cells. (**A**) Replication kinetics of indicated lentiviral strain in pre-activated and IFNα-treated (500 u/ml; dotted line) or untreated (solid line) human PBMCs up to 10 days post-infection. Infectious virus yield was quantified using a TZM-bl reporter assay. Shown is the mean of three individual donors, each tested in triplicates of infection ± SEM. (**B**) Average time needed to reach a replication peak of each virus group based on panel (A). Mean + SD. (**C**) Resistance to IFNα treatment based on panel (A). Mean + SD. *, *P* < 0.05; **, *P* < 0.01; calculated by Mann–Whitney test.

HIV-2 originated from and is genetically closely related to SIVsmm strains but is highly divergent from HIV-1, the main causative agent of AIDS. To compare the ability of HIV-1, HIV-2, and SIVsmm to evade human intrinsic immune responses, we analyzed their replication in the presence and absence of type I IFN in primary human PBMCs containing T lymphocytes [23]. To ensure that the results are representative of the lentiviral groups, we selected panels of five IMCs of HIV-1, including four primary transmitted-founder IMCs of subtypes B and C (CH77, CH58, THRO, and CH167), five HIV-2 IMCs including the patient-derived 7312A strain, and five SIVsmm strains representing diverse viral lineages (L1–L5). In the absence of IFNα, all viruses replicated efficiently, with HIV-2 strains reaching maximum virus production around day 6 and SIVsmm showing moderately delayed replication kinetics (Fig. 1A and B). HIV-1 strains replicated very efficiently also in the presence of IFN (2-fold reduction), while HIV-2 replication was inhibited by ∼5-fold (Fig. 1C). In comparison, SIVsmm strains were more strongly suppressed by type I IFN (32-fold reduction) and generally replicated poorly in its presence. Thus, SIVsmm exhibits lower replication fitness and immune evasion than HIV-1 and HIV-2, suggesting suboptimal adaptation to type I IFN-induced antiviral restriction factors.

### HIV-2 evolved ZAP resistance

ZAP is a broadly acting antiviral restriction factor that forms an antiviral complex with TRIM25 ubiquitin ligase and endoribonuclease KHNYN to degrade CpG-containing viral transcripts. Their expression is upregulated by IFN (Supplementary Fig. S1A) [3, 4], which suggests that the ZAP antiviral complex might contribute to the restriction of SIVsmm in human PBMCs.

**Figure 2.**
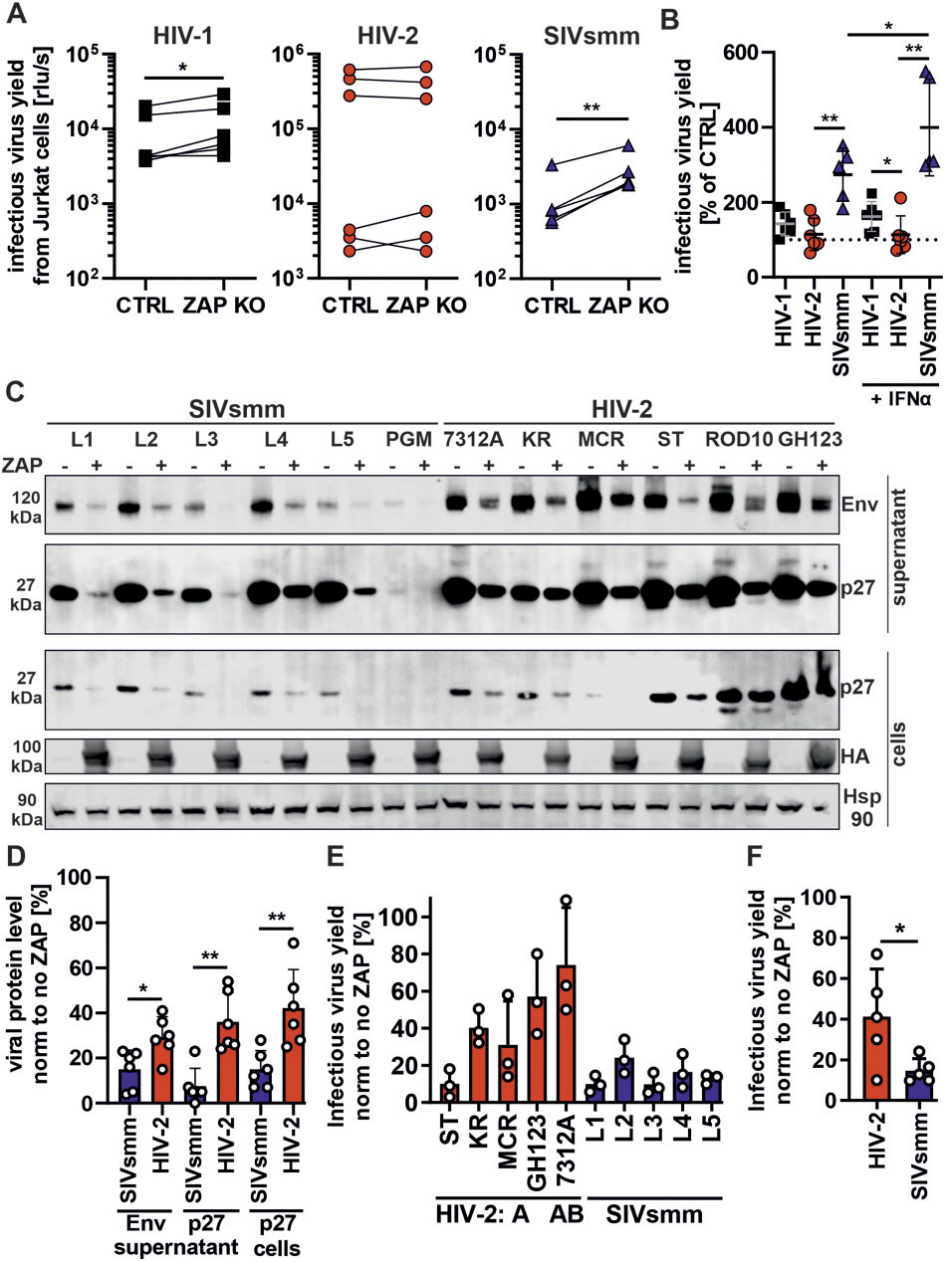
Inhibition of HIV-2 and SIVsmm by ZAP. (**A**) HIV-1 (square), HIV-2 (circle), and SIVsmm (triangle) infectious virus production in parental (CTRL) or ZAP knockout (KO) CCR5-expressing Jurkat cells 6 days after transduction. (**B**) Infectious virus yield % calculated by dividing virus yield in the absence (KO) and presence (CTRL) of ZAP, in the absence and presence of 500 u/ml IFNα treatment. (**C**) Western blot of HEK293T ZAP KO cells and their supernatants following the transfection of empty BFP vector control or HA-tagged ZAP and indicated infectious molecular clone of SIVsmm or HIV-2, and (**D**) its quantification. Hsp90 serves as loading control. (**E**) Infectious virus yield of HIV-2 and SIVsmm produced from HEK293T ZAP KO cells co-transfected with indicated infectious molecular clone and human HA-tagged ZAP. Values were normalized to infectious virus produced in the absence of ZAP expression. (**F**) Average infectious virus produced from HEK293T ZAP KO cells in the presence of ZAP overexpression based on (**E**). Infectious virus yield was quantified using a TZM-bl reporter assay. *N* = 3 + SD. *, *P* < 0.05; **, *P* < 0.01; calculated using Student’s *t*-test.

To determine the contribution of ZAP to IFN susceptibility of HIV-2 and SIVsmm, we used Jurkat CCR5 CRISPR–Cas9 ZAP KO cells [27]. This cell line was selected due to its resemblance to human T lymphocytes, the natural target cells of HIV. In addition to previously tested HIV-2 clones, we also included HIV-2 group A MCR strain, which is attenuated in primary human cells but replicates well in immortalized cell lines [54, 55]. The infection of control and ZAP KO Jurkat cells with HIV-1, HIV-2 and SIVsmm resulted in a detectable virus production of all strains (Fig. 2A and Supplementary Fig. S1B). ZAP KO increased infectious HIV-1 production by 50% on average, while HIV-2 was unaffected by ZAP (Fig. 2B). In contrast, ZAP KO enhanced SIVsmm infection by 200% in the absence of IFNα and up to 450% in the presence of IFNα treatment. Thus, ZAP restricts SIVsmm more efficiently than HIV-1 and HIV-2 in human T cells.

To ensure that these results were not biased by relatively low replication of SIVsmm strains in this system, we performed overexpression experiments in HEK293T ZAP KO cells [7]. For this, we used an expression plasmid encoding the long isoform of ZAP (ZAP-L), which is the most antivirally active isoform of ZAP [2, 56, 57]. Transfection of SIVsmm IMCs resulted in similar or slightly lower viral protein expression and infectious virus yield than HIV-2 (Supplementary Fig. S1C), in agreement with previous data [13, 23]. On average, ZAP reduced the p27 and Env protein production of SIVsmm by ∼90% but only by ∼65% in the case of HIV-2 (Fig. 2C and D). HIV-2 strains differed in ZAP sensitivity from being highly resistant (HIV-2 7312A, directly isolated from patient PBMCs) to as sensitive as SIVsmm (HIV-2 ST, passaged in cell lines) (Fig. 2E). On average, SIVsmm production was reduced to 2.7 times lower levels by ZAP than that of HIV-2 (Fig. 2F). Altogether, these results clearly suggest that HIV-2 evolved increased ZAP resistance during its adaptation to humans.

### HIV-2 evades ZAP despite higher CpG content in a species-independent manner

**Figure 3.**
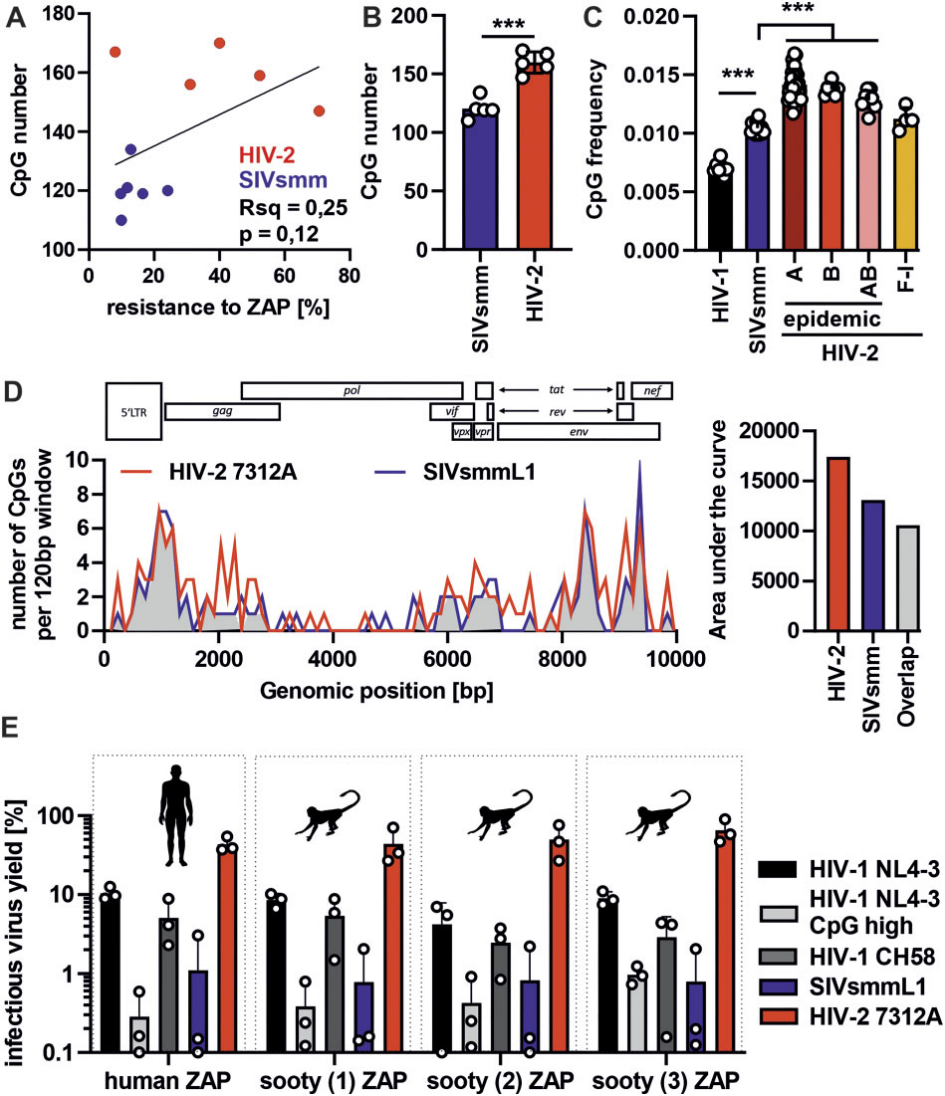
CpG number and distribution in HIV-2 and SIVsmm genomes and the antiviral activity of human and sooty ZAP orthologs. (**A**) Correlation between CpG number in HIV-2 and SIVsmm strain genomes and their resistance to ZAP overexpression (related to Fig. 2E). (**B**) Average CpG number in the tested HIV-1, HIV-2, and SIVsmm strains and (**C**) average CpG frequency in HIV-1, SIVsmm, and HIV-2 (groups A-I) full-genome sequences obtained from the Los Alamos HIV sequence database. (**D**) Left: distribution of CpGs in the aligned HIV-2 7312A and SIVsmm L1 genomes and overlap in CpG positions. ORF position in the HIV-2 genome is shown for reference above the graph. Right: Quantification of the area under the curve for HIV-2, SIVsmm and overlapping peaks. (**E**) Infectious virus yield of HIV-1, HIV-2, and SIVsmm co-transfected with 0.4 μg DNA of human and sooty mangabey ZAP in ZAP KO HEK293T cells. Infectious virus yield was quantified using a TZM-bl reporter assay. *N* = 3 + SD. ***, *P* < 0.001, calculated using the Mann–Whitney test.

CpG suppression plays a key role in the evasion of ZAP restriction by many viruses [1, 2, 7, 58]. For the set of HIV-2 and SIVsmm IMCs analyzed; however, high CpG numbers did not correlate with increased ZAP sensitivity and even showed the opposite trend (Fig. 3A). On average, HIV-2 strains had a significantly higher CpG content than SIVsmm and HIV-1 (Fig. 3B and C). Extended analysis of the 72 available full viral genome sequences from the Los Alamos HIV database confirmed that epidemic HIV-2 A and B strains show significantly higher CpGs frequencies than SIVsmm (Fig. 3C). In contrast, the CpG frequencies in rare HIV-2 F-I did not differ significantly from those of SIVsmm. Thus, epidemic HIV-2 strains evolved ZAP resistance despite an overall increase in CpG content. We have previously shown that the sensitivity of HIV-1 to ZAP is determined by the number of CpGs in the first 600 bp of the *env* ORF rather than the whole viral genome [13]. Thus, we compared the distribution of CpGs across HIV-2 and SIVsmm genomes to identify site-specific differences in CpG content. HIV-2 contained an increased number of CpGs throughout the genome (Fig. 3D). The location of CpGs in HIV-2 7312A substantially overlapped with those found in SIVsmmL1 (Fig. 3D). Furthermore, HIV-2 did not decrease ZAP expression levels or affect subcellular localization (Supplementary Fig. S2A–C) suggesting that it evades rather than directly counteracts ZAP.

ZAP orthologs from different vertebrate species exhibit varying degrees of activity and CpG-specificity [10, 59]. To determine if differences between human and sooty mangabeys ZAP activity could explain the high sensitivity of SIVsmm, we amplified ZAP from the cDNA of two captive sooty mangabeys. We obtained three ZAP variants and cloned them into an expression vector. The otherwise highly conserved RNA-binding domain (RBD) of sooty mangabey ZAP differed at three positions from human ZAP (Supplementary Fig. S3A). To determine the potential impact of these mutations on antiviral activity, we co-transfected HEK293T ZAP KO cells with ZAP and HIV-1, HIV-2, or SIVsmm proviruses. Sooty mangabey ZAP strongly inhibited the control CpG-enriched HIV-1 mutant virus and restricted HIV-1 and SIVsmm as efficiently as human ZAP, while HIV-2 7312A was generally less affected by either ortholog (Fig. 3E). Thus, sooty mangabey ZAP is as active as its human ortholog and HIV-2 evolved resistance to ZAP proteins of both host species.

### HIV-2 RNA is bound by ZAP but resistant to its antiviral effects due to changes in the 3′ half of the genome

**Figure 4.**
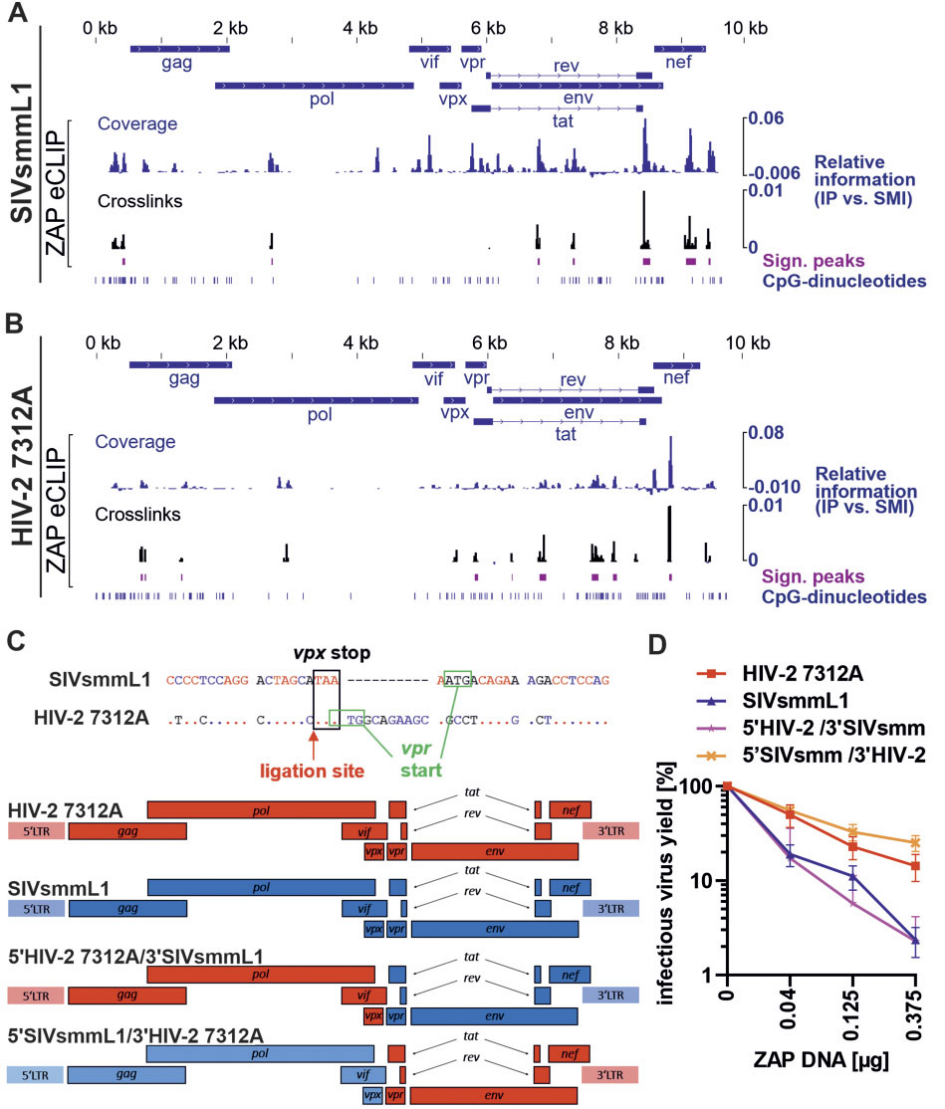
Analysis of ZAP-binding regions in HIV-2 and SIVsmm RNA and mapping of the determinants of ZAP resistance. (**A**) Alignment of HA-ZAP eCLIP data to SIVsmmL1 and (**B**) HIV-2 7312A genomic RNA. Relative coverage information in IP versus SMI is calculated at each position and displayed along the viral genome. ZBSs significantly enriched relative to SMI are shown. For each significantly enriched ZBS, crosslinking events were extracted (relative information in IP versus SMI). Location of CpG-dinucleotides is displayed. (**C**) Position of ligation of the half HIV-2/ half SIVsmm virus chimeras and schematic of generated chimeric proviral constructs showing HIV-2 (red) and SIVsmm (blue) fragments. (**D**) Sensitivity of those chimeras to ZAP overexpression in HEK293T ZAP KO cells. Infectious virus yield was quantified using a TZM-bl reporter assay; *N* = 3 + SD.

RNA binding is essential for the antiviral activity of ZAP [3, 56]. To compare ZAP binding events across the HIV-2 and SIVsmm RNA, we performed enhanced crosslinking and immunoprecipitation in combination with RNA sequencing analysis (eCLIP-seq) in HEK293T ZAP KO cells co-transfected with HIV-2 or SIVsmm proviruses and ZAP. We normalized the signal observed in ZAP immunoprecipitations relative to a SMI control and observed seven significantly enriched peak regions (FDR < 0.05, log 2-fold change > 0.5; Table 2), all of which contained at least one CpG (Fig. 4A). In the case of HIV-2 RNA, we identified nine significantly enriched ZBSs (FDR < 0.05, log2 fold change > 0.5; Table 2) of which five overlapped with CpG positions (Fig. 4B). In both viruses, most ZBSs localized to the 3′ genome half (downstream of the *vpr* gene). To analyze the impact of this region on ZAP resistance, we generated chimeras between the resistant HIV-2 7312A and sensitive SIVsmmL1. To avoid changes within the viral ORFs, we selected the *vpx/vpr* gene border (Fig. 4C) to generate IMCs carrying 5′ and 3′ genome halves of HIV-2 or SIVsmm, respectively. Chimeric virus containing the 3′ half of the HIV-2 genome was more resistant to ZAP overexpression than the virus containing the 3′ half of SIVsmm (Fig. 4D and Supplementary Fig. S4A). Thus, ZAP sensitivity is determined by the 3′ viral genome half, which contains most of the ZBSs.

**Table 2.** Significant peaks identified by eCLIP

	Start	End	Count IP	Count SM	noPeak. IP	no Peak. SM	log2FC (IP/SM)	Odds ratio	*P*-value	p.adj	Score	log pval
**SIVsmmL1 ZAP-binding peaks**												
1	416	454	851	42	146 869	10 870	0.55	1.50	0.004	0.0315	1.50	2.37
2	2707	2723	269	3	147 451	10 909	2.32	6.63	5.872e-6	1.145 e-4	3.94	5.23
3	6797	6830	492	20	147 228	10 892	0.80	1.82	0.002	0.028	1.55	2.53
4	7339	7368	673	29	147 047	10 883	0.73	1.72	0.001	0.016	1.79	2.85
5	8410	8528	2887	143	144 833	10 769	0.57	1.50	3.660e-7	1.684 e-5	4.77	6.44
6	9080	9226	2573	125	145 147	10 787	0.60	1.53	5.761e-7	1.684 e-5	4.77	6.24
7	9416	9447	755	32	146 965	10 880	0.76	1.75	5.535e-4	0.008	2.09	3.26
**HIV-2 7312A ZAP-binding peaks**												
1	695	724	3117	59	1 863 543	97 117	1.44	2.75	4.771 e-20	1.049 e-18	17.98	19.32
2	762	777	1350	30	1 865 310	97 146	1.18	2.34	8.810 e-8	1.210 e-6	5.92	7.06
3	1318	1334	765	16	1 865 895	97 160	1.23	2.49	2.270 e-5	2.480 e-4	3.61	4.64
4	5801	5857	9830	346	1 856 830	96 830	0.56	1.48	1.495 e-14	2.739 e-13	12.56	13.83
5	6367	6376	1018	32	1 865 642	97 144	0.69	1.66	0.002	0.012	1.90	2.79
6	6798	6899	17 477	638	1 849 183	96 538	0.51	1.43	5.384 e-21	1.480 e-19	18.83	20.26
7	7598	7694	17 556	408	1 849 104	96 768	1.16	2.25	1.669 e-76	9.177 e-75	74.04	75.78
8	7917	7981	14 717	491	1 851 943	96 685	1.44	2.75	4.771 e-20	1.049 e-18	17.98	19.32
9	8774	8821	19 858	291	1 846 802	96 885	1.18	2.34	8.810 e-8	1.210 e-6	5.92	7.06

### HIV-2 evades ZAP restriction by an *env*-independent mechanism

**Figure 5.**
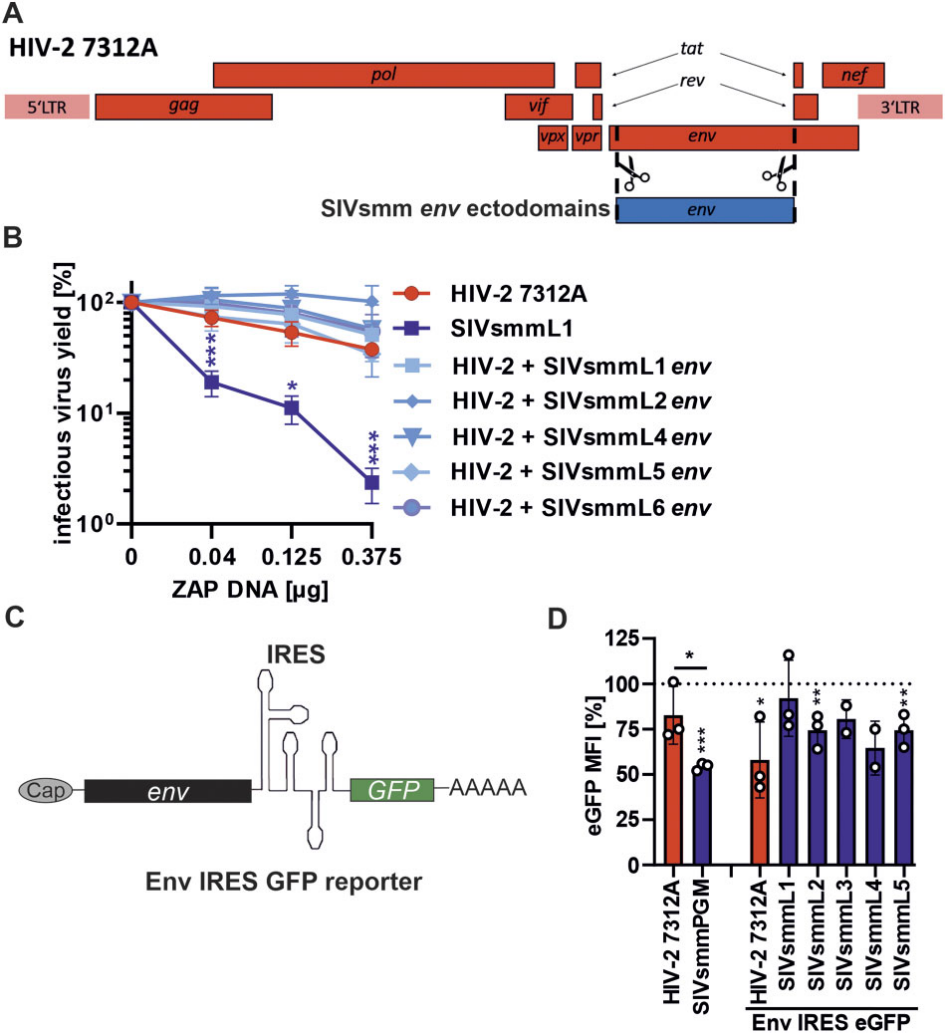
Impact of ZAP on HIV-2 Env chimeras with SIVsmm Env ectodomains and viral env transcripts. (**A**) Schematic of HIV-2 7312A-based Env ectodomain chimeras and (**B**) their sensitivity to ZAP overexpression compared to wild type HIV-2 7312A and SIVsmmL1 strains. Infectious virus yield was quantified using a TZM-bl reporter assay. (**C**) Schematic of env IRES eGFP reporter and (**D**) flow cytometry results showing eGFP MFI in HEK293T ZAP KO cells co-transfected with ZAP or ZAP mutant without RNA-binding domain (ZAP delta RBD; negative control; 100%) and either IRES eGFP reporter viruses or pCG Env IRES eGFP reporter variants. *N* = 3 + SD. *, *P* < 0.05; **, *P* < 0.01; ***, *P* < 0.001, calculated using Student’s *t*-test.

The largest ORF found in the 3′ half of the HIV-2 and SIVsmm genomes encodes for the Env protein. We have previously shown that CpG content at the 3′ end of the *env* gene determines ZAP sensitivity of HIV-1 [13]. However, CpG levels in the *env* genes of HIV-2 and SIVsmm did not correlate with ZAP restriction (Supplementary Fig. S4B). To determine if the *env* coding region contributed to the difference in ZAP sensitivity of HIV-2 and SIVsmm, we utilized published HIV-2 7312A recombinants encoding different SIVsmm Env ectodomains [17] (Fig. 5A). HIV-2 WT and all chimeras were resistant to ZAP overexpression, while SIVsmmL1 was strongly inhibited (Fig. 5B). To additionally determine the contribution of the full-length *env* ORF region, we utilized bicistonic *env* IRES eGFP reporter constructs (Fig. 5C). In this system, ZAP targeting of the *env* region in the RNA transcript reduces GFP expression. Comparison of GFP signal of the control HIV-2 7312A-IRES-GFP and SIVsmm PGM-IRES-GFP reporter viruses in the absence and presence of ZAP showed significant inhibition of SIVsmm but not HIV-2 7312A, indicating that the IRES eGFP cassette itself did not change ZAP phenotype (Fig. 5D, left). In contrast, ZAP decreased GFP signal of HIV-2 7312A and two SIVsmm *env*-IRES-eGFP reporters, and there was no significant difference between them (Fig. 5D, right). These results show that the determinants of ZAP sensitivity of the HIV-2/SIVsmm lineage differ from that of HIV-1 and are *env*-independent.

### HIV-2′s Nef coding region contributes to ZAP resistance

**Figure 6.**
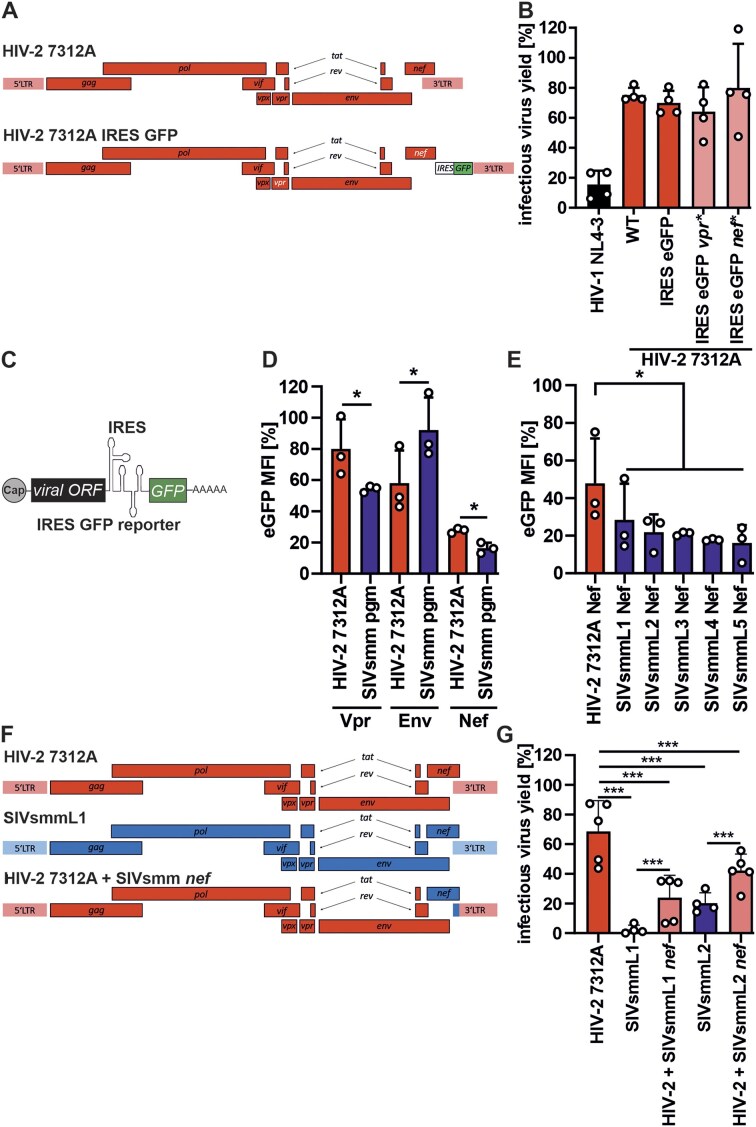
Mapping of determinants of ZAP resistance using chimeric HIV-2/SIVsmm clones. (**A**) Diagram showing the genomes of the HIV-2 7312A (WT) and the HIV-2 7312A IRES GFP reporter virus in which the expression of vpr or nef gene has been abrogated by introducing two consecutive premature stop codons within the viral ORFs. (**B**) effect of ZAP overexpression (0.3 μg) on HIV-2 7312A wild-type, reporter and mutants containing premature stop codons (*) in vpr and nef ORFs in HEK293T ZAP KO cells. Values were normalized to infectious virus yield obtained in the presence of co-expression of 0.3 μg control vector and each virus strain. (**C**) Schematic of vpr/env/nef IRES eGFP reporters and (**D**) eGFP MFI in HEK293T ZAP KO cells co-transfected with indicated IRES eGFP reporters and ZAP or inactive ZAP delta RBD construct (negative control; 100%) and (**E**) Nefs from HIV-2 7312A or SIVsmm L1-L5 (**F**). (F) Schematic of nef chimeric proviral constructs showing HIV-2 (red) and SIVsmm (blue) fragments and (**G**) sensitivity of chimeric viruses to ZAP overexpression (0.4 μg) relative to WT HIV-2 and SIVsmm strains. Infectious virus yield was quantified using a TZM-bl reporter assay. *N* = 3–5 + SD. *, *P* < 0.05; **, *P* < 0.01; ***, *P* < 0.001, calculated using Student’s *t*-test.

The accessory proteins of HIV are known to antagonize several antiviral restriction factors [60]. Therefore, we tested whether the HIV-2 accessory proteins Vpr and Nef, encoded by the 3′ genome half, counteract ZAP. HIV-2 7312A IRES eGFP virus with premature stop codons in *vpr* or *nef* remained resistant to ZAP (Fig. 6A and B), indicating that these proteins do not act as ZAP antagonists.

To examine whether HIV-2 and SIVsmm *vpr* and/or *nef* encoding regions are responsible for differential ZAP inhibition of the two viruses, we utilized an extended panel of bicistronic IRES eGFP reporters (Fig. 6C). The fluorescence of the GFP reporter expressed from the same mRNA as the viral ORF was measured in the presence of wild-type (WT) human ZAP or inactive ZAP mutant carrying a deletion of the four zinc-fingers (ZAP delta RBD; set to 100%). WT ZAP reduced eGFP expression from mRNAs containing SIVsmm *vpr* and *nef* genes significantly more efficiently than the corresponding HIV-2 constructs (Fig. 6D). The reporter containing SIVsmm *nef* ORF was more efficiently inhibited (85% decrease) by ZAP than that containing *vpr* (45% decrease). Further analyses demonstrated that bicistronic IRES-eGFP constructs containing *nef* alleles from diverse SIVsmm variants are also more sensitive to ZAP restriction than the construct containing HIV-2 7312A *nef* (Fig. 6E).

To confirm the impact of the *nef* coding region on viral resistance to ZAP, we generated additional chimeric HIV-2 7312A/SIVsmm constructs (Fig. 6F). Unfortunately, SIVsmmL1 chimera containing HIV-2 *nef* gene was poorly infectious precluding meaningful analyses (Supplementary Fig. S4A, right panel). Introduction of SIVsmmL1 and SIVsmmL2 *nef* region increased HIV-2’s sensitivity to ZAP by 30%–50% (Fig. 6G). These results show that the *nef* ORF is an important determinant of the differential ZAP susceptibility of HIV-2 and SIVsmm.

### The *nef*/U3 LTR overlap region determines ZAP resistance of HIV-2

**Figure 7.**
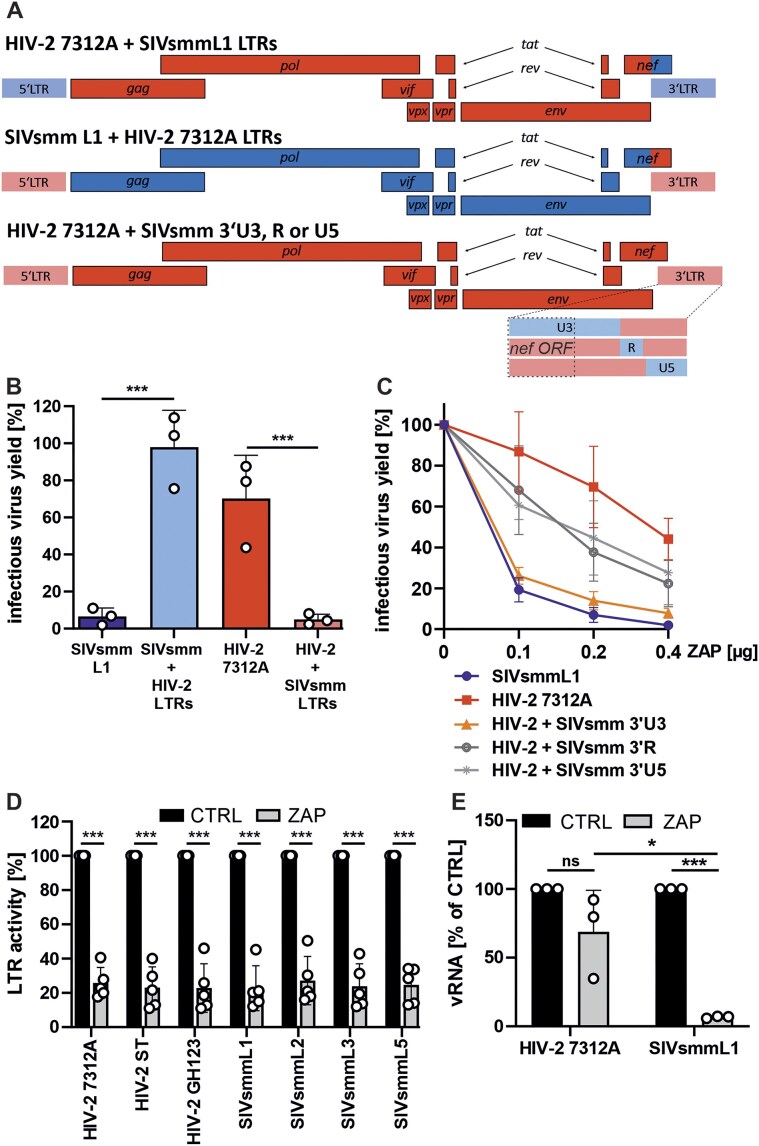
Impact of LTRs on HIV-2′s resistance to ZAP. (**A**) Schematic of the generated chimeric proviruses in which either both the 5′ and 3′ LTRs are exchanged, or only the U3, R, or U5 region of the 3′LTR. SIVsmm L1 regions are shown in blue while HIV-2 7312A regions are shown in red. (**B**) Impact of ZAP overexpression on the infectious virus yield of 5′+3′ LTR virus chimeras or (**C**) 3′ LTR U3, R, and U5 chimeras. Infectious virus yield was quantified using a TZM-bl reporter assay. (**D**) Activity of luciferase reporter constructs expressed from HIV-2 or SIVsmm 5′ LTR promoter in the presence and absence of ZAP overexpression. (**E**) Impact of ZAP overexpression on vRNA levels in transfected HEK293T ZAP KO cells. *N* = 3–4 + SD. *, *P* < 0.05; ***, *P* < 0.001; calculated using Student’s *t*-test.

In the SIVsmm/HIV-2 lineage, the *nef* ORF overlaps with the U3 region of the 3′LTR by ∼350 bp (Supplementary Fig. S5). The U3 region is duplicated at the 5′ and 3′ ends in the proviral DNA, but is only found at the 3′ end of the genomic and subgenomic viral RNAs [61, 62]. To define a potential contribution of this region to ZAP sensitivity, we swapped the entire viral LTRs or its individual 3′ U5, R and U3 parts between HIV-2 7312A and SIVsmm L1 (Fig. 7A). The exchange of both LTRs completely reversed the ZAP phenotypes of HIV-2 and SIVsmm (Fig. 7B). However, it also reduced basal infectious virus production by one to two orders of magnitude even in the absence of ZAP (Supplementary Fig. S6A). The exchange of the 3′ U3 or U5 regions also significantly attenuated HIV-2 7312 by 1.5- and 3.5-fold, respectively, while mutation of the 3′R region had no significant impact (Supplementary Fig. S6B). The analysis of these 3′LTR chimeras showed that the exchange of the U3 region sensitized HIV-2 to ZAP, while R and U5 regions had an intermediate effect (Fig. 7C).

During reverse transcription of lentiviral RNA into DNA, the 3′ LTR is used as a template for the reconstruction of full length 5′ LTR DNA, which drives the transcription of viral genes after integration. To determine whether ZAP suppresses LTR-driven transcription, we used a reporter assay in which luciferase expression is driven by the full-length 5′LTR of HIV-2 or SIVsmm [63]. Since the U3 region is not transcribed into mRNA in this system, its role is limited to transcriptional regulation. ZAP overexpression significantly reduced reporter activity for both LTRs, likely due to the CpG-rich sequence of the luciferase mRNA (Fig. 7D). Importantly, the type of LTR did not influence ZAP sensitivity, supporting the notion that ZAP targets the 3′U3 region of vRNA at the post-transcriptional level.

ZAP binding causes translational repression of vRNA and/or induces post-transcriptional degradation of the bound vRNA by endoribonucleases ([8, 64–66]). To determine if ZAP binding of SIVsmm RNA leads to efficient degradation of viral transcripts, we measured vRNA levels. ZAP overexpression potently reduced the SIVsmm RNA containing the nef/U3 overlap region while having only a modest and non-significant impact on the corresponding HIV-2 transcript levels (Fig. 7E). Thus, the 3′ U3 region which partially overlaps with the *nef* ORF is a major determinant of HIV-2’s ZAP resistance and its targeting in SIVsmm results in vRNA degradation.

### An adaptation in the U3 region of HIV-2 that mediates ZAP resistance promotes viral fitness in primary human T cells

**Figure 8.**
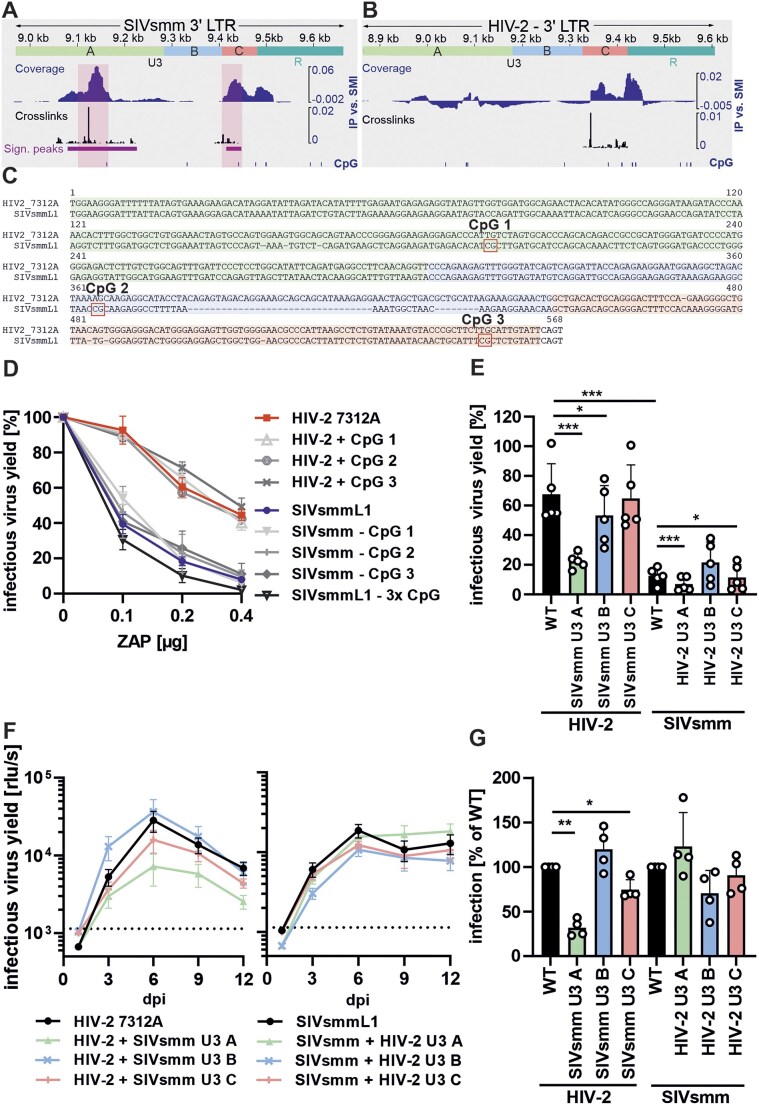
Mapping of U3 region responsible for HIV-2’s resistance to ZAP and its impact on viral fitness. (**A**) ZAP eCLIP data aligned to SIVsmmL1 and (**B**) HIV-2 7312A 3′ LTR region (based on Fig. 4A and B). (**C**) Alignment of the HIV-2 7312A and SIVsmm L1 U3 sequences showing the location of CpGs that are exclusively found in SIVsmmL1 (CpG 1, 2, and 3) and U3 regions A, B, and C. (**D**) Infectious virus yield of HIV-2 and SIVsmm CpG mutants or (**E**) chimeric viruses carrying exchanges in the 3′ U3 region A, B, or C, in HEK293T ZAP KO cells transfected with ZAP. *N* = 3–5 + SD. (**F**) Replication kinetics of chimeric HIV-2 and SIVsmm viruses carrying exchanges in the 3′ U3 region A, B, or C in human PBMCs over 12 days. (**G**) Normalized infectious virus yield of indicated U3 mutants as compared to WT HIV-2 7312A or SIVsmmL1 (100%) based on area under the curve analysis of data shown in the panel. (F) Infectious virus yield was quantified using a TZM-bl reporter assay. Mean of four independent donors + SD.

The iCLIP analysis of viral RNA bound by ZAP (Fig. 4AB and ) identified two binding regions within the SIVsmm L1 U3 (Fig. 8A). In comparison, these regions were not bound (ZAP-binding site 1; ZBS1) or only weakly bound (ZBS2) in HIV-2 7312A RNA (Fig. 8B). Sequence alignments revealed three CpGs located in SIVsmm L1 but not in the HIV-2 7312A U3, two of which were found in the proximity of the ZBSs (Fig. 8C). To test whether these sites are targeted by ZAP and responsible for differential restriction of both viruses, we generated mutants that were depleted (SIVsmm) or enriched (HIV-2) in CpGs at these sites. The introduction or removal of these CpGs did not have a significant effect on viral infectivity in the absence or presence of ZAP (Fig. 8D and Supplementary Fig. S6C).

To map the changes that contribute to ZAP resistance, we divided the U3 into three regions (Fig. 8C). Regions A and C contained the ZBS1 and 2 identified by iCLIP. Region B contained no ZBS and was the most divergent region between HIV-2 and SIVsmm (Supplementary Fig. S7A). Previous SHAPE structure probing of the SIVmac239 RNA [67], a virus closely related to and derived from SIVsmm, indicated that region B can fold a stable hairpin structure (Supplementary Fig. S7B), which according to *in silico* RNA fold predictions does not form in HIV-2 (Supplementary Fig. S7C and D). To determine if changes within these regions contribute to ZAP resistance, we generated HIV-2/SIVsmm chimeras with exchanges in the U3 regions A, B, and C (Supplementary Fig. S8A and B). The introduction of SIVsmm U3 region A into HIV-2 led to a 3-fold decrease in ZAP resistance, while the reverse chimera in SIVsmm backbone remained highly ZAP sensitive (Fig. 8E). HIV-2 with U3 region B of SIVsmm also showed a minor loss of ZAP resistance, while the corresponding SIVsmm region B mutant showed a minor increase in resistance (Fig. 8E). In comparison, the exchange of the U3 region C had no effect on either virus. Thus, the U3 region A, containing ZBS1, is the main determinant of HIV-2’s ZAP resistance.

To determine if the identified U3 region A associated with ZAP resistance contributes to HIV-2 fitness in primary human cells, we examined the replication of the chimeric HIV-2 and SIVsmm viruses in pre-activated human PBMCs (Fig. 8F). Exchange of the U3 region A reduced the cumulative infectious virus production of HIV-2 7312A by 3.5-fold (Fig. 8G). Meanwhile, the exchange of the other two U3 regions had no (region B) or only modest (region C) attenuating effect on HIV-2 replication. The corresponding SIVsmmL1-based chimeras showed no significant differences in replication.

Together, these results show that ZAP binds CpGs in SIVsmm U3 LTR and that HIV-2 7312A evolved changes in the U3 region promoting ZAP escape and replication fitness through a mechanism independent of CpG-depletion.

### HIV-2’s ZAP resistance maps to a Nef region that lost SERINC5 and tetherin antagonism

**Figure 9.**
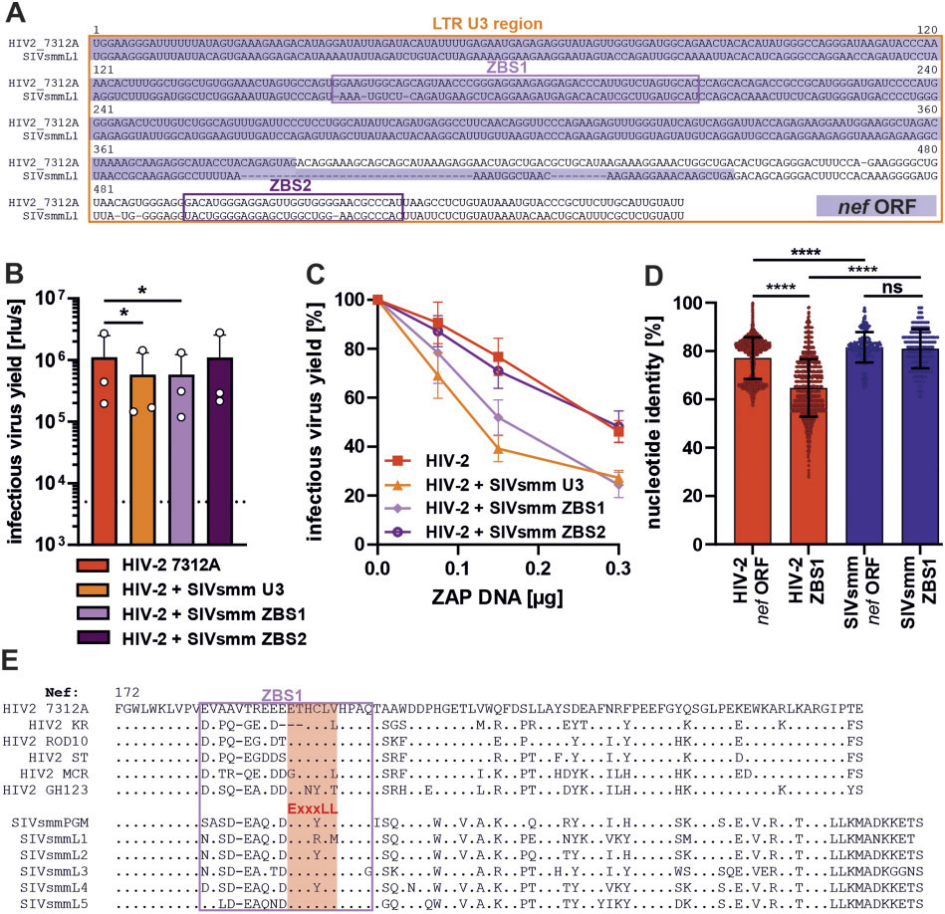
Evolutionary changes in HIV-2 U3/nef region contributing to ZAP resistance. (**A**) Alignment of the HIV-2 7312A and SIVsmm L1 U3 sequences showing the location of two ZAP-binding regions identified by RNA CLIP peaks (CLIP peak 1 and CLIP peak 2). The nef open reading frame (ORF) is shown. (**B**) Infectious virus yield of HIV-2 viruses containing 3′ SIVsmm U3 region, CLIP peak 1 region, and CLIP peak 2 region in HEK293T cells, and (**C**) reduction in infectious virus yield of HIV-2 mutants in the presence of ZAP overexpression. *N* = 3 + SD. *, *P* < 0.05; calculated using Student’s *t*-test. (**D**) Nucleotide identity of HIV-2 and SIVsmm nef ORF and CLIP peak 1 area. Each dot represents pair-wise identity value calculated for HIV-2 and SIVsmm sequences available from Los Alamos HIV sequence database and error bars show standard deviation. ****, *P* < 0.0001; ns = not significant; calculated using Mann–Whitney *U*-test. (**E**) Amino acid alignment of HIV-2 and SIVsmm Nef proteins showing region encoded by the RNA CLIP peak 1 region. Nef's “ExxxLL” a canonical leucine-based AP-binding motif important for tetherin and SERINC5 counteraction is shown.

The 3′ LTR U3 region largely overlaps with the *nef* ORF (Fig. 9A). The ZBS1 is found within the overlap region and might influence Nef’s function, while the ZBS2 is found downstream of the *nef* ORF in the non-coding LTR region. To determine if changes within ZBS1 contribute to ZAP resistance of HIV-2, we introduced the two ZBS regions of SIVsmmL1 into the HIV-2 7312A clone. The exchange of the whole 3′ LTR U3 region and the exchange of ZBS1 resulted in a 2-fold decrease in infectious virus yield in the absence of ZAP (Fig. 9B), as well as similar level of sensitization of HIV-2 to ZAP (Fig. 9C). In contrast, the exchange of ZBS2 had no significant effect on infectious virus yield in the absence (Fig. 9B) or presence of ZAP overexpression (Fig. 9C), which is consistent with the phenotype of the HIV-2 U3 region C mutant (Fig. 8E). To determine if the ZBS1 region has undergone purifying or diversifying selection in the natural hosts of SIVsmm and HIV-2, we compared the conservation of the whole *nef* ORF and ZBS1 region in the available HIV-2 and SIVsmm nucleotide sequences from the Los Alamos HIV database (Fig. 9D). HIV-2 *nef* showed a significantly higher level of sequence variability than SIVsmm. The nucleotide conservation of the SIVsmm *nef* ORF and ZBS1 was similar, indicating a lack of specific evolutionary selection acting on this region. However, HIV-2 showed a significantly lower level of nucleotide sequence conservation of the ZBS1 compared to the whole ORF, indicating a diversifying selection acting on this *nef* region.

While the Nef protein itself does contribute to HIV-2′s ZAP resistance (Fig. 6B), changes associated with the evolution of ZAP resistance might influence Nef function. To assess potential consequences of this HIV-2 adaptation, we mapped the localization of the ZBS1 in the HIV-2 and SIVsmm Nef amino acid sequences. The region contributing to ZAP resistance overlaps with the C-loop and ExxxLL motif of Nef (Fig. 9E). This region is critical for the ability of SIVsmm Nef to counteract tetherin and SERINC5, while HIV-2 Nefs have largely lost these functions [17, 19, 68]. In summary, the mutations contributing to ZAP’s resistance of HIV-2 were associated with a decrease in functional constraints of viral Nef due to the loss of tetherin and SERINC5 counteraction.

## Discussion

ZAP restricts the replication of many RNA and DNA viruses, often in an IFN-dependent manner [1]. The ability of ZAP to preferentially target foreign RNA transcripts relies on its selective binding to CpG dinucleotides [7], which represent the least abundant type of dinucleotide in the vertebrate genomes [69]. HIV-1 and many other RNA viruses strongly suppress their CpG content and thus at least partially escape ZAP’s activity [7, 58, 70–73]. Here, we show that epidemic HIV-2 strains evolved resistance to ZAP despite increasing their CpG content by ∼33% compared to their zoonotic ancestor, SIVsmm. This evasion of ZAP is mediated by changes in the *nef*/U3 LTR region that promote HIV-2 replication fitness in primary human T cells. This region has undergone substantial diversification during the spread of HIV-2 in humans, likely facilitated by reduced functional constraints on the overlapping *nef* gene. Our results indicate that ZAP contributes to IFN-mediated response against SIVsmm in human cells and show the versatility of primate lentiviruses in evading ZAP-mediated antiviral immunity. While the host ZAP activity has been shown to correlate with the levels of CpG suppression in primate lentiviruses [59], our findings suggest that evolution of an efficient ZAP evasion strategy allows viruses to tolerate increased genomic CpG levels. Thus, ZAP presents a barrier for zoonotic viruses, and effective ZAP evasion can reduce the pressure for CpG suppression.

The link between viral CpG suppression and ZAP evasion is well established [7, 13, 58, 70–72, 74–76]. However, the determinants of ZAP sensitivity are complex and include not only the CpG number, but also their localization, context and local RNA structure [13, 74, 76]. We have previously shown that CpG frequency in the 5′ end of the *env* gene determines the sensitivity of primary HIV-1 strains to ZAP [13]. However, HIV-2 *env* plays no role in its resistance to ZAP, which underscores the divergent evolutionary paths taken by HIV-1 and HIV-2 in adapting to human immune defences and evading ZAP. In case of SIVsmm, ZAP efficiently decreased both Env and Gag levels, which are expressed from independent, spliced and unspliced viral mRNA, respectively. The broad viral transcript targeting by ZAP can be explained by the fact that all HIV-2 and SIVsmm RNA splicing products contain the 3′ U3 LTR region. Analyses of chimeric HIV-2/SIVsmm recombinants, RNA levels and iCLIP revealed that this RNA region is specifically targeted by ZAP. Furthermore, our findings show that the *nef* ORF contributes to ZAP evasion due to its overlap with 3′ U3 LTR, but the Nef protein does not directly antagonize ZAP. Surprisingly, removal of the only CpG found in the mapped ZAP-binding region had no impact on SIVsmm’s sensitivity to ZAP (Fig. 8D). Furthermore, only five out of nine ZBSs in HIV-2 RNA contain a CpG dinucleotide. It remains to be clarified how non-CpG motifs in viral RNAs influence ZAP’s binding and restriction.

Many Nef functions, such as downmodulation of CD4, class I MHC and CD28, are conserved between HIV-2 and SIVsmm [17, 77, 78]. However, HIV-2 switched from Nef-mediated SERINC5 and tetherin antagonism to Env-mediated evasion [17, 19, 68]. Both of these functions depend on residues in and adjacent to the ExxxLL endocytosis motif in SIVsmm Nef. A deletion in the cytoplasmic tail of human tetherin disrupts the interaction with SIV Nefs, and HIV-2 evolved Env as tetherin antagonists [17]. Similarly, HIV-2 Nefs lost their ability to efficiently counteract SERINC5 during human adaptation [19] but changes in Env mediate resistance [79, 80]. We found that the nucleotide changes that confer ZAP resistance to HIV-2 map to the part of the U3 region that encodes amino acid residues critical for tetherin and SERINC5 antagonism by SIVsmm Nef. Our data suggest that the loss of these activities reduced the functional constrains on HIV-2 Nef and facilitated changes in the U3 region allowing efficient evasion of ZAP restriction.

ZAP contributes to the restriction of many animal-infecting RNA viruses [81–86]. Here, we show that ZAP contributes to IFN-mediated restriction of SIVsmm in primary human T cells and most likely restricts its replication at the post-transcriptional level, leading to efficient degradation of viral RNA by cofactors such as KHNYN and XRN1 [8, 65, 86, 87]. Therefore, ZAP constitutes a part of the interspecies barrier protecting humans from zoonotic pathogens. ZAP targets the UTRs not only of SIVsmm but also several viral and host mRNAs [7, 88–93]. The untranslated regions often form complex structures that recruit RNA processing enzymes and other cellular factors [94]. It remains to be established if highly structured RNAs are commonly recognized and targeted by ZAP and whether pathogens with increased CpG content other than HIV-2 utilize an CpG-independent ZAP evasion strategy.

While our results identify ZAP as an effective inhibitor of SIVsmm replication in human T cells during IFN-response activation, it is not the only antiviral restriction factor that represents a cross-species barrier to this zoonotic virus. For example, it has been shown that SIVsmm is less efficient at counteracting human APOBEC3F [23], IFI16 [95], and GBP5 than HIV-2 [23, 95–97], suggesting that the latter had to evolve multiple innate immune evasion strategies to spread in humans. Apart from ZAP, type I IFN upregulates the expression of hundreds of other ISGs [98–100], most of which have never been studied in the context of HIV-2 or SIVsmm restriction. Deciphering the contribution of all individual human ISGs to SIVsmm restriction would be an ambitious and time-consuming task that lies beyond the scope of the current study. Nevertheless, the generated chimeric HIV-2/SIVsmm infectious molecular clones will be highly useful for further studies investigating the innate immune mechanisms limiting SIVsmm cross-species transmission and spread in humans.

We found that the U3 region of HIV-2 accumulated changes during its human-specific adaptation that contribute to replicative fitness and mediate ZAP’s resistance. The corresponding 5′ part of the U3 region of SIVsmm was significantly bound by ZAP, and its introduction into HIV-2 7312A increased ZAP sensitivity. It remains to be clarified how the structure of this RNA region affects ZAP recognition and how changes in this sequence affect the replication and ZAP sensitivity of other groups of HIV-2 and lineages of SIV. In agreement with our findings, it has been reported that while the Sp1 and NF-kB transcription binding sites are generally conserved, the U3 region of patient-derived HIV-2 isolates has extremely high genomic variability [101]. Considering the presence of this region in both unspliced and spliced RNA products, it is surprising that so little is known about the physiological role of the U3 region upstream of the major Sp1 and NF-κB transcription factor binding sites [101]. Our finding that this poorly-studied RNA region enables evasion of an IFN-induced antiviral restriction factor uncovers a new role of the HIV-2 U3.

SIVsmm replicates in human cells but is highly sensitive to IFN-induced innate responses. The contribution of ZAP to this restriction might seem counter-intuitive, as sooty mangabeys express a highly active ZAP, and SIVsmm has co-evolved with its host much longer than HIV-2 with humans. However, the difference in the antiviral immune response activation can may explain this phenomenon: sooty mangabeys infected by SIVsmm have high viral loads but do not show chronic immune activation and inflammation leading to a progressive CD4 + T cell loss and immunodeficiency [18, 43, 102–104]. This is likely a result of a long pathogen-host co-evolution and the lack of fully functional innate immune sensors in sooty mangabeys [102, 105]. In contrast, HIV-2 infection in humans is characterized by high levels of immune activation and IFN production that upregulates the expression of ZAP and its cofactor TRIM25 [2, 9, 82, 84]. We show that epidemic HIV-2 had to evolve increased fitness and IFN resistance during its adaptation to humans. A part of this human-specific adaptation was mediated by mutations in the U3/*nef* region that increase replicative fitness in human T cells and allow evasion of ZAP through a CpG-independent mechanism. This adaptation reduced the selective pressure acting against CpGs in the HIV-2 genome, leading to a substantial accumulation of these rare dinucleotide over the course of its evolution.

In conclusion, we show that ZAP contributes to the IFN-mediated restriction of SIVsmm in human T cells and how HIV-2 evolved to evade it. By demonstrating that HIV-2′s resistance to ZAP is mediated by a CpG-independent mechanism involving the U3/*nef* overlap region, we identified a novel ZAP evasion mechanism, that requires further molecular characterization. It remains to be clarified whether ZAP evasion contributes to the fitness of HIV-2 *in vivo*. It will be interesting to test if untranslated RNA regions of other viruses are also targeted by ZAP and if other successful zoonotic viral pathogens evolved similar evasion mechanisms. Thus, further studies on the interplay between ZAP and viral RNAs will provide insights into the virus-host arms race and the mechanisms shaping viral evolution.

## Supplementary Material

gkaf826_Supplemental_File

## Data Availability

The data generated by iCLIP has been deposited to GEO repository under the identifier GSE290608. The primary data underlying this article as well as generated resources will be shared on a reasonable request to the corresponding author.
