# Supplementary material for: HIV-2 evades restriction by ZAP through adaptations in the U3 LTR region despite increased CpG levels
Source: Nucleic Acids Res. 2025 Aug 30;53(16):gkaf826. doi: 10.1093/nar/gkaf826 (PMC12397911; doi:10.1093/nar/gkaf826)
Supplement: gkaf826_Supplemental_File [file gkaf826_supplemental_file.pdf]

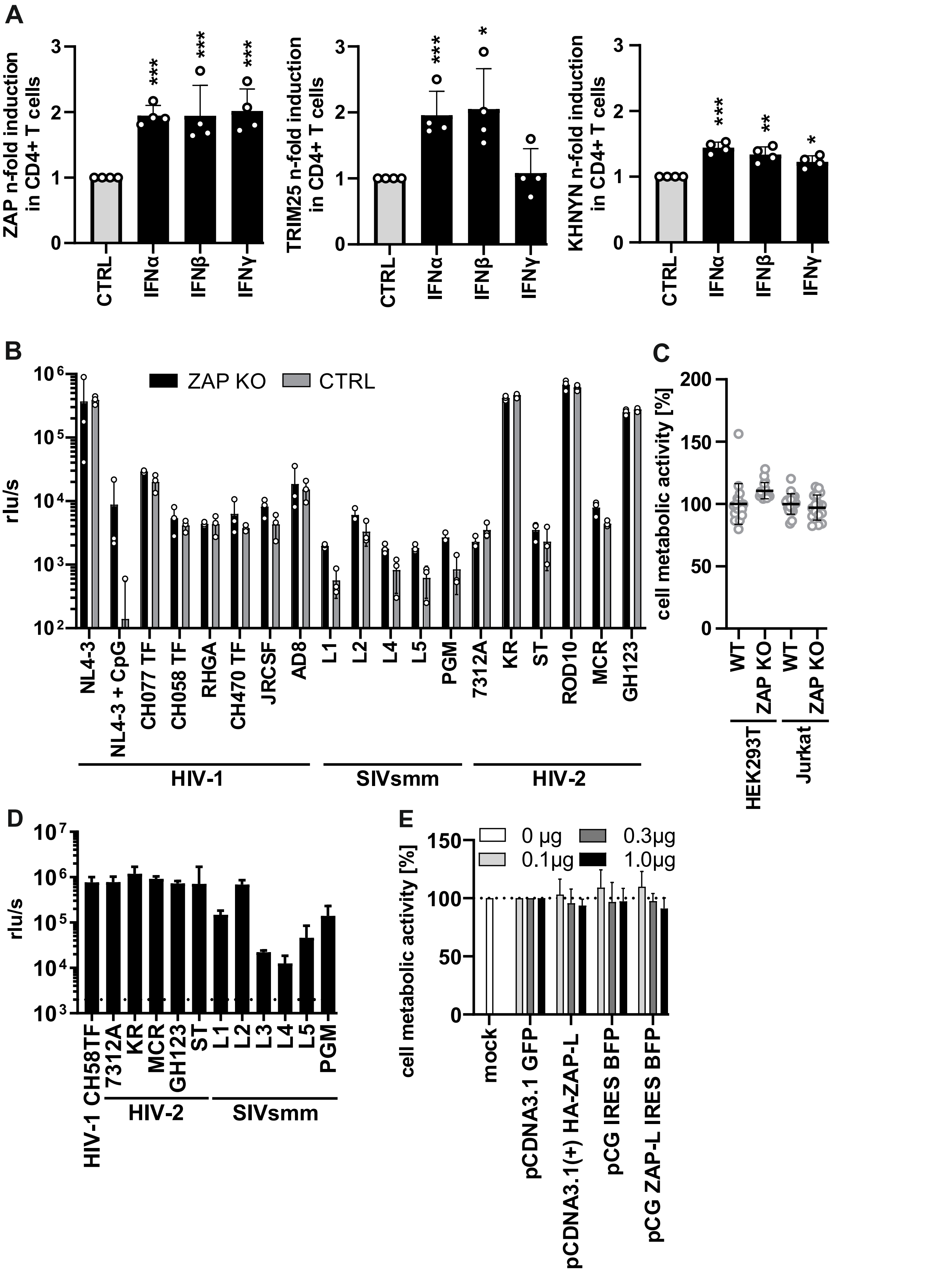
A
ZAP n-fold induction in CD4+ T cells
TRIM25 n-fold induction in CD4+ T cells
KHNYN n-fold induction in CD4+ T cells
CTRL
IFNα
IFNβ
IFNγ
B
ZAP KO
CTRL
rlu/s
NL4-3
NL4-3 + CpG
CH077 TF
CH058 TF
RHGA
CH470 TF
JRCSF
AD8
L1
L2
L4
L5
PGM
7312A
KR
ST
ROD10
MCR
GH123
HIV-1
SIVsmm
HIV-2
C
cell metabolic activity [%]
WT
ZAP KO
HEK293T
Jurkat
D
rlu/s
HIV-1 CH58TF
7312A
KR
MCR
GH123
ST
L1
L2
L3
L4
L5
PGM
HIV-2
SIVsmm
E
cell metabolic activity [%]
0 µg
0.1µg
0.3µg
1.0µg
mock
pCDNA3.1 GFP
pCDNA3.1(+) HA-ZAP-L
pCG IRES BFP
pCG ZAP-L IRES BFP

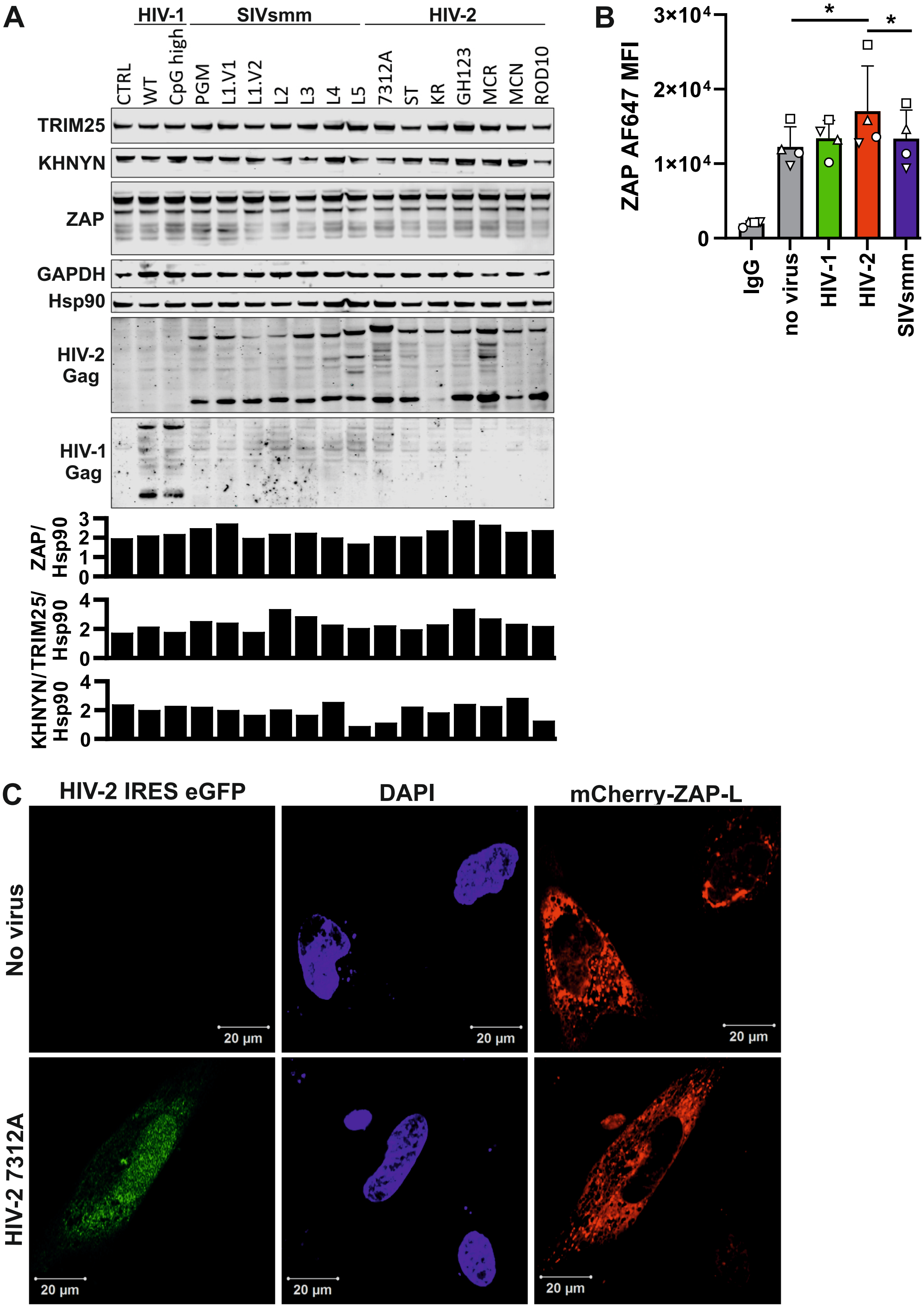
A
HIV-1
SIVsmm
HIV-2
CTRL
WT
CpG high
PGM
L1.V1
L1.V2
L2
L3
L4
L5
7312A
ST
KR
GH123
MCR
MCN
ROD10
TRIM25
KHNYN
ZAP
GAPDH
Hsp90
HIV-2 Gag
HIV-1 Gag
ZAP/ Hsp90
TRIM25/ Hsp90
KHNYN/ Hsp90
B
ZAP AF647 MFI
3×10⁴
2×10⁴
1×10⁴
0
*
*
IgG
no virus
HIV-1
HIV-2
SIVsmm
C
HIV-2 IRES eGFP
DAPI
mCherry-ZAP-L
No virus
HIV-2 7312A
20 µm

Figure S3

Kmiec et al., 2025

A

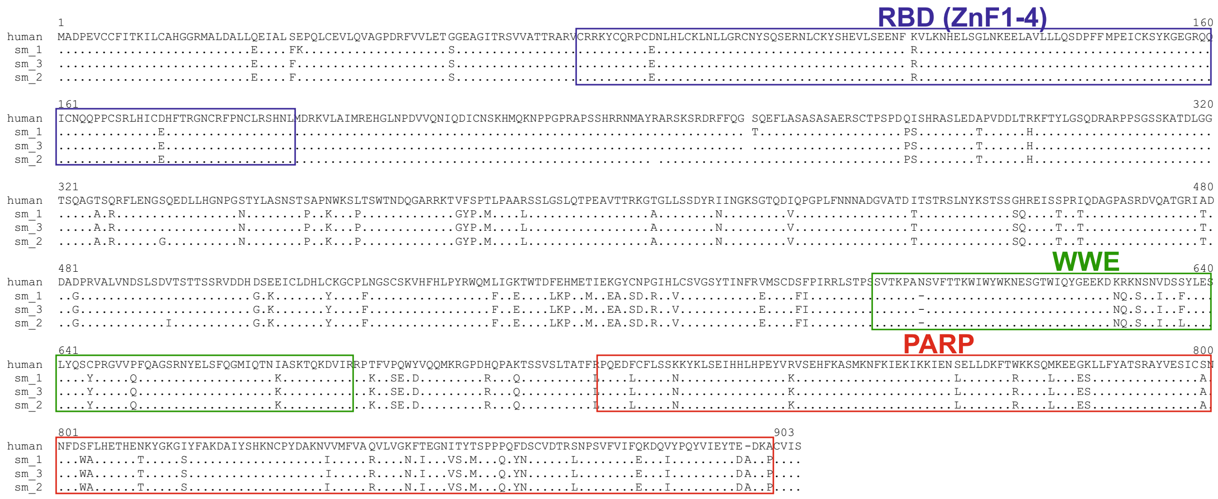

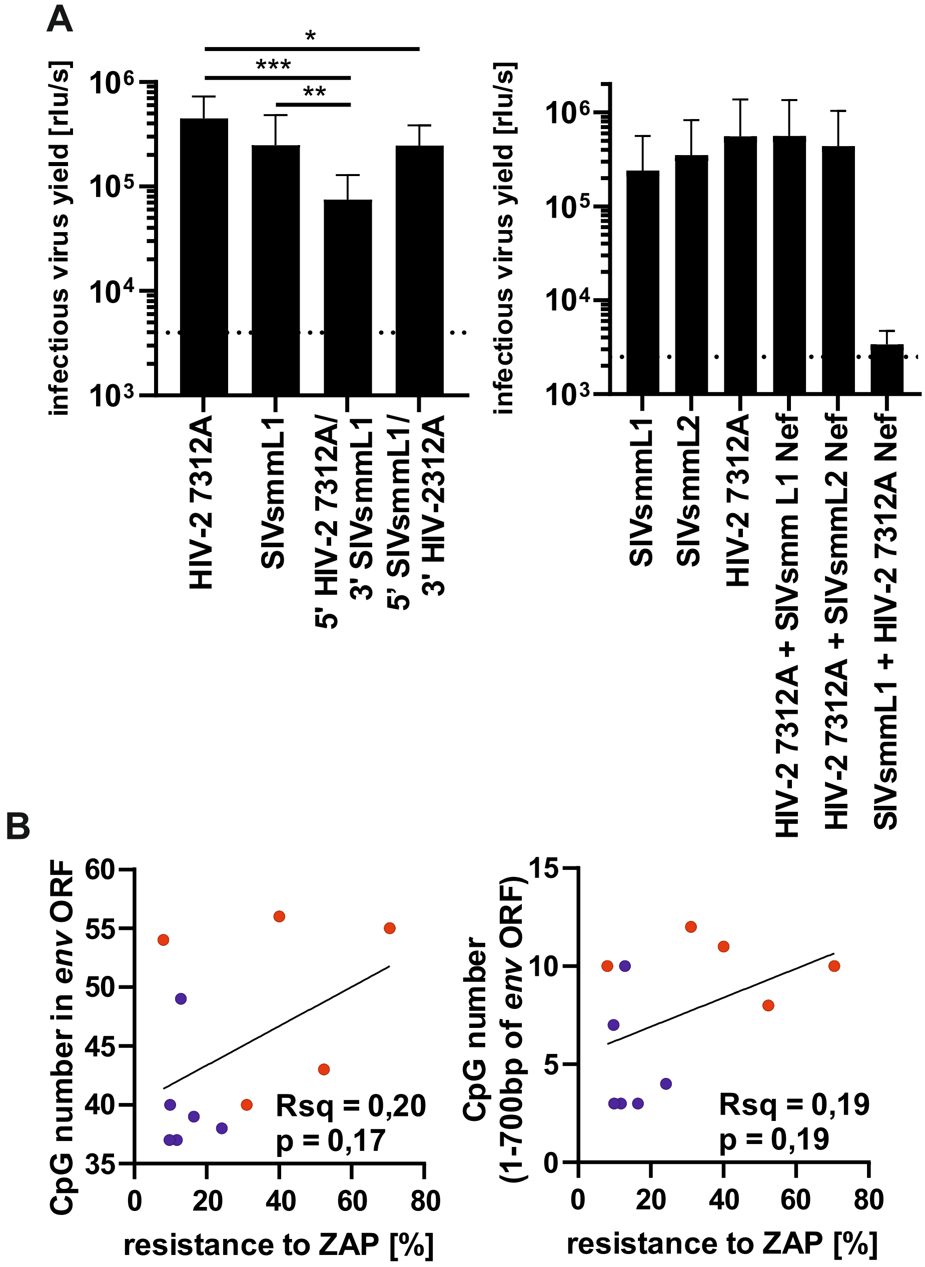
A
infectious virus yield [rlu/s]
HIV-2 7312A
SIVsmmL1
5' HIV-2 7312A/ 3' SIVsmmL1
5' SIVsmmL1/ 3' HIV-2312A
*
***
**
SIVsmmL2
HIV-2 7312A + SIVsmm L1 Nef
HIV-2 7312A + SIVsmmL2 Nef
SIVsmmL1 + HIV-2 7312A Nef
B
CpG number in env ORF
resistance to ZAP [%]
Rsq = 0,20
p = 0,17
CpG number (1-700bp of env ORF)
Rsq = 0,19
p = 0,19

```
        1                                                                                                 100
7312A   TGGAAGGGATTTTTTATAGTGAAAGAAGACATAGGATATTAGATACATATTTTGAGAATGAGAGAGGTATAGTTGGTGGATGGCAGAACTACACATATGG
  PGM   TGGAAGGGATTTATTACAGTGAGAGAAGACATAGAATCTTAGACATATACTTAGAAAAGGAAGAAGGCATCACCCCAGATTGGCAGAATTACACATCAGG
   L1   TGGAAGGGATTTATTACAGTGAAAGGAGACATAAAATATTAGATCTGTACTTAGAAAAGGAAGAAGGAATAGTACCAGATTGGCAAAATTACACATCAGG
   L2   TGGAAGGGATTTATTACAGTGATAGGAGACATAAAATATTAGATCTGTACTTAGAAAAGGAAGAAGGAATAGTACCAGATTGGCAAAATTACACATCAGG
   L3   TGGAAGGGATATATTACAGTGAAAGAAGGCATAAAATATTAGATTTATACCTAGAAAAAGAAGAAGGGATCATTCCTGATTGGCAGAATTACACAAAAGG
   L4   TGGAAGGGATTTATTACAGTGAGAGGAGACATAGAATATTAGACATATACCTAGAAAAGGAAGAAGGAATAATACCTGATTGGCAGAATTACACCTCAGG
   L5   TGGAAGGGATTTATTACAATCAGAGAAGACATAGAATACTAGACATTTACTTAGAAAAGGAGGAAGGAATCATCCCTGACTGGCAGAATTACACATCAGG

        101                                                                                               200
7312A   GCCAGGGATAAGATACCCAAAACACTTTGGCTGGCTGTGGAAACTAGTGCCAGTGGAAGTGGCAGCAGTAACCCGGGAGGAAGAGGAGACCCATTGTCTA
  PGM   ACCAGGAATCAGATACCCAATGTTCTTTGGCTGGCTATGGAAATTGGTCCCAGTAAGTGCCTCAGATGAAGCTC---AAGAAGACGAGACACATTACTTA
   L1   GCCAGGAACCAGATATCCTAAGGTCTTTGGATGGCTCTGGAAATTAGTCCCAGTAAATGTCTCAGATGAAGCTC---AGGAAGATGAGACACATCGCTTG
   L2   GCCAGGAATCAGATATCCTAAGGTCTTTGGATGGCTCTGGAAATTAGTCCCAGTAAATGTCTCAGATGAAGCTC---AGGAAGATGAGACACATTGTTTG
   L3   GCCAGGGATAAGATACCCAATGACGTTTGGGTGGCTATGGAAGCTAGTGCCAGTAAATGTATCAGATGAGGCAG---AAACAGATGAGACCCACTGTCTG
   L4   GCCAGGAATAAGATATCCAATGTGCTTTGGATGGTTGTGGAAATTAGTACCAGTAGATGTCTCGGATGAGGCAC---AAGAAGATGAGACACACTATCTG
   L5   ACCAGGAATAAGATACCCAATGATGTTCGGATGGCTGTGGAAACTAGTCCCAGTAGAGGTCTTAGATGAGGCAC---AGAATGATGAGACACATTGTCTG

        201                                                                                               300
7312A   GTGCACCCAGCACAGACCGCCGCATGGGATGATCCCCATGGGGAGACTCTTGTCTGGCAGTTTGATTCCCTCCTGGCATATTCAGATGAGGCCTTCAACA
  PGM   GTGCATCCAGCTCAGATATCCCAGTGGGATGATCCCTGGGGAGAGGTTCTGGCATGGAAGTTTGATTCACAACTAGCCTACAGATATGAGGCTTTCATTA
   L1   ATGCATCCAGCACAAACTTCTCAGTGGGATGACCCCTGGGGAGAGGTATTGGCATGGAAGTTTGATCCAGAGTTAGCTTATAACTACAAGGCATTTGTTA
   L2   ATGCATCCAGCACAAATTTCTCAGTGGGATGACCCCTGGGGAGAGGTATTGGCATGGAAGTTTGATCCAGAGTTAGCTTACAATTATAAGGCATTTATTG
   L3   GTACATCCGGCAGGGACAAGCAAGTGGGATGATCCGTGGGGTGAAACCCTAGCATGGAGATTTGATCCAACACTGGCTCATAGCTATGAAGCATTCATTA
   L4   GTGCATCCTGCACAGACAAGTCAGTGGAATGACCCATGGGGAGAAGTTCTAGCTTGGAAGTTTGATCCCACATTAGCTTATACTTATGAGGCATTTGTTA
   L5   GTGCATCCAGCACAGACGGGCCAATGGGATGACCAGTGGGGAGAGGTCTTGGCATGGAAGTTTGACCCCACTCTGGCTTATACCTATGAGGCATATATTA

                                                                                              nef STOP
        301                                                                                               400
7312A   GGTTCCCAGAAGAGTTTGGGTATCAGTCAGGATTACCAGAGAAGGAATGGAAGGCTAGACTAAAAGCAAGAGGCATACCTACAGAGTAGACAGGAAAGCA
  PGM   GACACCCAGAAGAGTTTGGCAGTAAGTCAGGCTTGTCAGAGGAAGAGGTAAAGAGAAGGCTAACCGCAAGAGGCCT------------------------
   L1   AGTACCCAGAAGAGTTTGGTAGTATGTCAGGATTGCCAGAGGAAGAGGTAAAGAGAAGGCTAACCGCAAGAGGCCT------------------------
   L2   AGCACCCAGAAGAGTTTGGTAGTATGTCAGGCTTGTCAGAGGAAGAGGTGAAGAGAAGGCTAACCGCAAGAGGCCT------------------------
   L3   GATACCCAGAGGAGTTTGGATGGAGCTCAGGGCTGTCACAGGAAGAGGTTGAGAGAAGGCTAACCGCAAGAGGCCT------------------------
   L4   AATATCCAGAAGAATTTGGTAGCAAGTCAGGCTTGTCAGAGGAAGAGGTTAAGAGAAGGCTAACCGCAAGAGGCCT------------------------
   L5   AGTACCCAGAAGAGTTTGGGGACAAGTCAGGCTTGTCAGAGGAAGAGGTTAAGAGAAGGCTAACCGCAAGAGGCCT------------------------

                                                       nef STOP
        401                                                                                               500
7312A   GCAGCATAAAGAGGAACTAGCTGACGCTGCATAAGAAAGGAAACTGGCTGACACTGCAGGGACTTTCCAGAAGGGGCTGTAACAGTGGGAGGGACATGGG
  PGM   ----TTTAAAAA-----TGGCTGAC--------AAGAAGGAAACAAGCTGAGACAGCAGGGACTTTCCACAAAGGG-GATGTTACGGGGAGGTACTGGGG
   L1   ----TTTAAAAA-----TGGCTAAC--------AAGAAGGAAACAAGCTGAGACAGCAGGGACTTTCCACAAAGGG-GATGTTATGGGGAGGTACTGGGG
   L2   ----TTTAAAAA-----TGCCTGAC--------AAGAAGGAAACAAGCTGAGACAGCAGGGACTTTCCACAAAGGG-GATGTTATGGGGAGGTACCGGGG
   L3   ----TTTAAAGA-----TGGCTGAC--------AAGGGAGGAAACAGCTGAGACAGCAGGGACTTTCCACAAGGGGCTGTAACA-GGGGAGGTACTTGGG
   L4   ----CTTAAAAA-----TGGCTGAC--------AAGAAGGAAACTAGCTGAGACAGCAGGGACTTTCCACAA-------------GGGGATGTTATGGGG
   L5   ----TTTAAAAA-----TGGCTGAC--------AAGAAGGAAACTAGCTGAGACTGCAGGGACTTTCCACAGTGGGAGGCATCAATGGGAGGTAACTGGG

        501                                                      558
7312A   AGGAGTTGGTGGGGAACGCCCATTAAGCCTCTGTATAAATGTACCCGCTTCTTGCATT
  PGM   AGGAGCTGGCTGG-AACGCCCACTTATTTTCTGTATAAATACAACTGCATTTCGCTCT
   L1   AGGAGCTGGCTGG-AACGCCCACTTATTCTCTGTATAAATACAACTGCATTTCGCTCT
   L2   AGGAGCTGGCTGG-AACGCCCACTTATTCTCTGTATAAATACAACTGCATTTCGCTCT
   L3   AGGAGCTGGTGGGGAACGCCCA-TTATTCTCTGTATAAATGTAGCTGCTGAACGCTCT
   L4   AGGAGCTGGTCGGGAACGCCCACTTTCTTGATGTATAAATACAACTGCATTTCGCTCT
   L5   AGGTACTGGTGGGGAACGCCCATTTATTCTCTGTATAAATACAACCGCTTGTCGCTCT
```

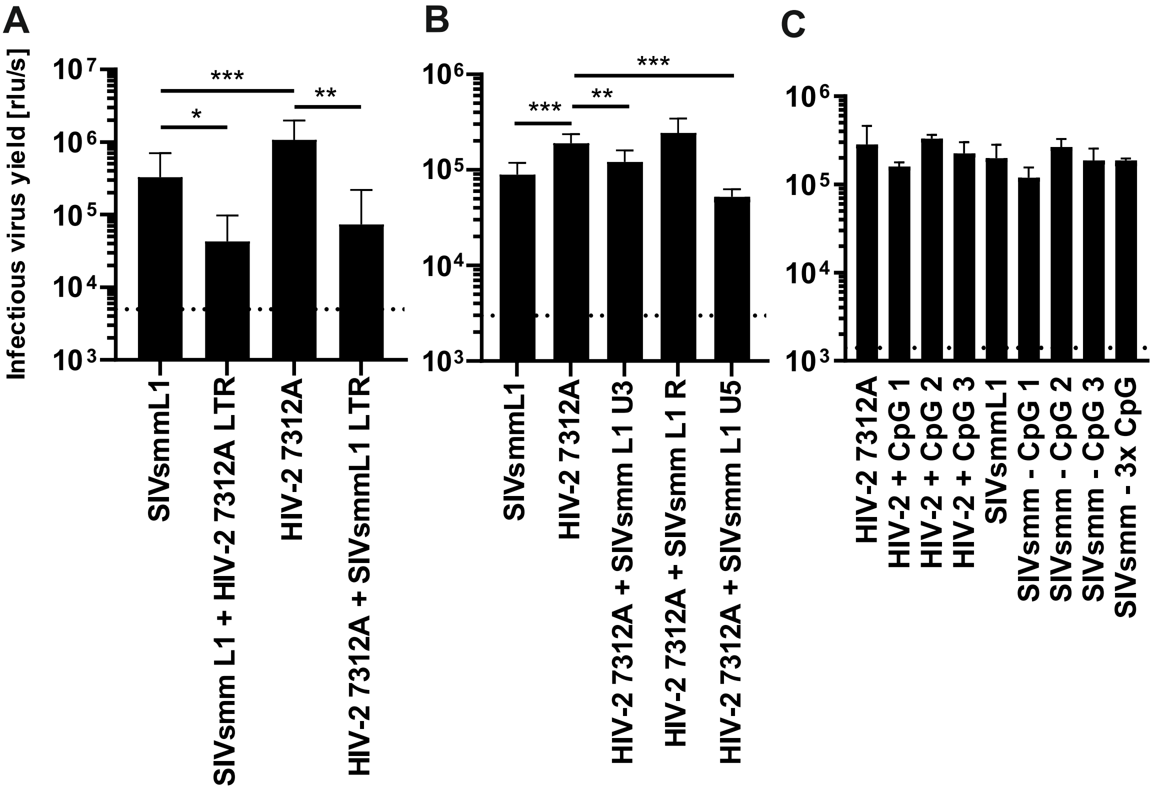
A
B
C
Infectious virus yield [rlu/s]
SIVsmmL1
SIVsmm L1 + HIV-2 7312A LTR
HIV-2 7312A
HIV-2 7312A + SIVsmmL1 LTR
HIV-2 7312A + SIVsmm L1 U3
HIV-2 7312A + SIVsmm L1 R
HIV-2 7312A + SIVsmm L1 U5
HIV-2 + CpG 1
HIV-2 + CpG 2
HIV-2 + CpG 3
SIVsmm - CpG 1
SIVsmm - CpG 2
SIVsmm - CpG 3
SIVsmm - 3x CpG

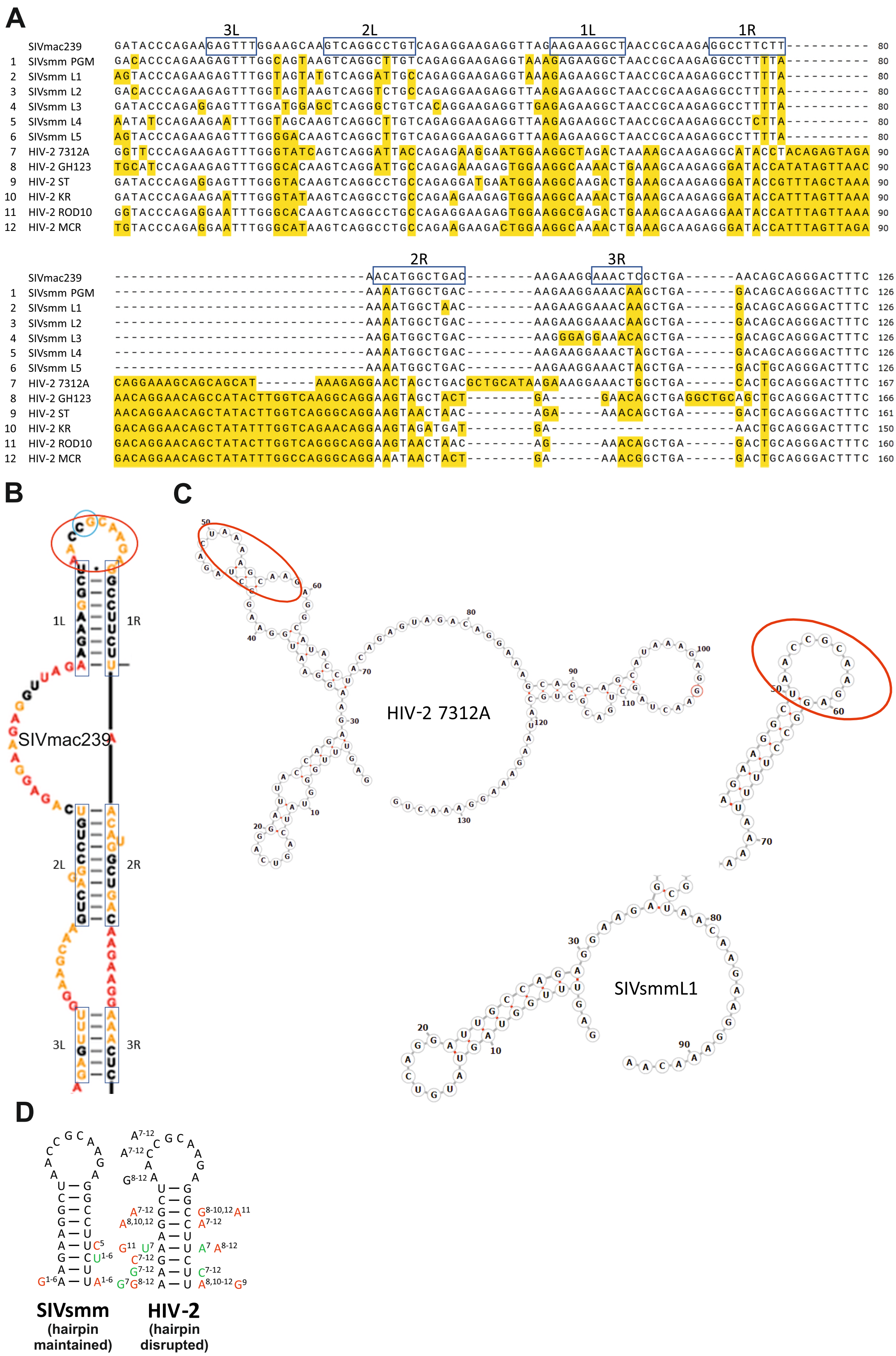
A
3L
2L
1L
1R
2R
3R
SIVmac239
1 SIVsmm PGM
2 SIVsmm L1
3 SIVsmm L2
4 SIVsmm L3
5 SIVsmm L4
6 SIVsmm L5
7 HIV-2 7312A
8 HIV-2 GH123
9 HIV-2 ST
10 HIV-2 KR
11 HIV-2 ROD10
12 HIV-2 MCR
B
C
SIVmac239
HIV-2 7312A
SIVsmmL1
D
SIVsmm
(hairpin maintained)
HIV-2
(hairpin disrupted)

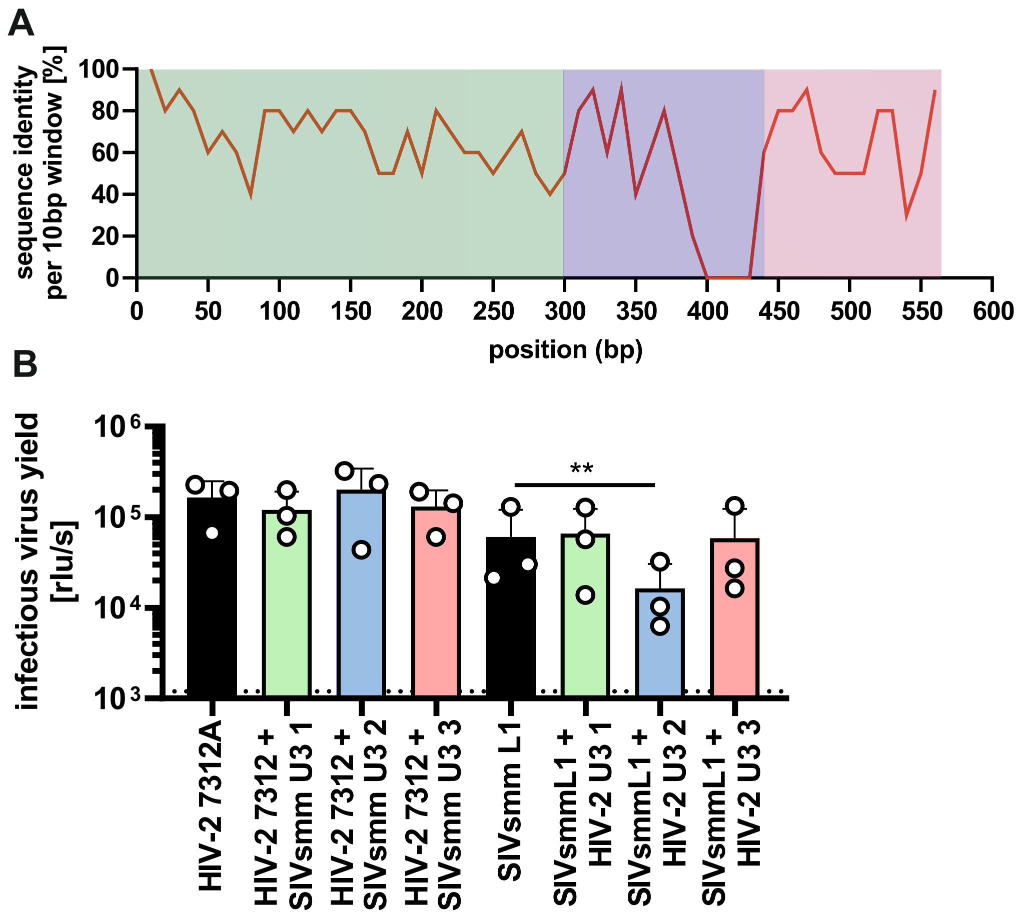
A
sequence identity per 10bp window [%]
100
80
60
40
20
0
0 50 100 150 200 250 300 350 400 450 500 550 600
position (bp)
B
infectious virus yield [rlu/s]
10^6
10^5
10^4
10^3
**
HIV-2 7312A
HIV-2 7312 + SIVsmm U3 1
HIV-2 7312 + SIVsmm U3 2
HIV-2 7312 + SIVsmm U3 3
SIVsmm L1
SIVsmmL1 + HIV-2 U3 1
SIVsmmL1 + HIV-2 U3 2
SIVsmmL1 + HIV-2 U3 3

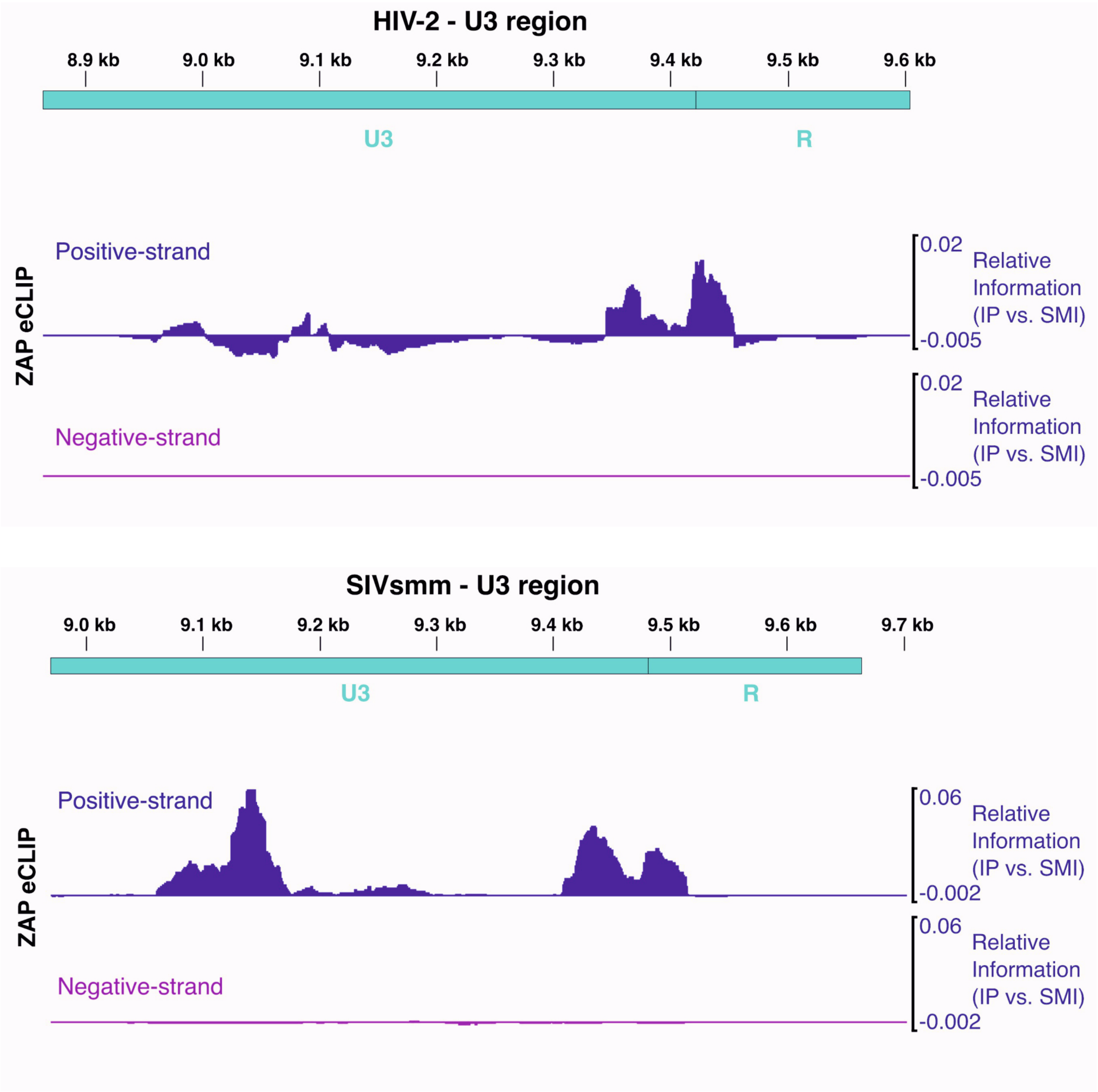
HIV-2 - U3 region
8.9 kb
9.0 kb
9.1 kb
9.2 kb
9.3 kb
9.4 kb
9.5 kb
9.6 kb
U3
R
ZAP eCLIP
Positive-strand
0.02
-0.005
Relative Information (IP vs. SMI)
Negative-strand
0.02
-0.005
Relative Information (IP vs. SMI)
SIVsmm - U3 region
9.0 kb
9.1 kb
9.2 kb
9.3 kb
9.4 kb
9.5 kb
9.6 kb
9.7 kb
U3
R
ZAP eCLIP
Positive-strand
0.06
-0.002
Relative Information (IP vs. SMI)
Negative-strand
0.06
-0.002
Relative Information (IP vs. SMI)

Supplementary figure legends:

Figure S1. (A) Related to Figure 1 and 2. *Expression levels of ZAP and its cofactors TRIM25 and KHNYN in primary human PBMCs 2 days after stimulation with 500u/ml type I IFN (α, β) or 200u/ml type II IFN(γ) measured by flow cytometry. Each dot represents one donor. (B) Related to Figure 2. Raw infectivity values of HIV-1, HIV-2 and SIVsmm in transduced Jurkat CCR5 cells quantified using a TZM-bl reporter assay. (C) Effect of ZAP KO on cell metabolic activity/viability in HEK293T and Jurkat cells. (D) Raw infectivity values of HIV-1, HIV-2 and SIVsmm in HEK293T ZAP KO cells quantified using a TZM-bl reporter assay and (E) effect of increasing doses of control and ZAP expression vectors on transfected HEK293T ZAP KO cell metabolic activity/viability 3 days post-transfection. N=3 +/- SD. *, $p < 0.05$; **, $p < 0.01$; ***, $p < 0.001$ calculated using Student's t-test.*

Figure S2. *Related to Figure 3. (A) Western blot of HEK293T cells transfected with indicated HIV-1, SIVsmm or HIV-2 provirus, and quantification of endogenous expression levels of ZAP, TRIM25 and KHNYN normalized to the Hsp90 housekeeping gene. (B) Expression levels of ZAP in pre-activated human PBMCs isolated from 4 donors, transduced with HIV-1, HIV-2 or SIVsmm and stained 3 days later with AF649-conjugated anti-ZAP and FITC-conjugated anti-p24 (Gag) antibodies. *, $p < 0.05$ calculated using paired Student's t-test. (C) Confocal microscopy images of HeLa cells co-transfected with HIV-2 IRES eGFP and mCherry-tagged ZAP and stained with nuclear stain (DAPI).*

Figure S3. *Related to Figure 3 and 4. (A) Protein alignment of human and sooty mangabey ZAP variants cloned from PBMC cDNA. RNA-binding domain containing 4 zinc-fingers (ZnF), WWE domain and catalytically inactive PARP domain are shown. Dots indicate amino acid conservation.*

Figure S4. *Related to Figure 4-6. (A) Raw infectivity values of chimeric HIV-2/SIVsmm half mutants and nef exchange mutant proviruses transfected into HEK293T ZAP KO cells. N=3 +SD. (B) Lack of significant correlation between CpG env number and ZAP resistance of HIV-2 (blue) and SIVsmm (red).*

Figure S5. *Related to Figure 6. DNA alignment of U3 LTR regions of HIV-2 7312A and SIVsmmL1-L5. CpGs are highlighted in green and the end of nef ORF is indicated by a black box.*

Figure S6. *Related to Figure 6. (A) Raw infectivity values of chimeric HIV-2/SIVsmm LTR mutant proviruses and (B) 3'LTR U3, R and U5 exchange mutants and (C) U3 CpG mutants in transfected into HEK293T ZAP KO cells.* N=3-5 + SD; **, p < 0.05; **, p< 0.01; ***, p<0.001 calculated using Student's t-test.*

Figure S7. Related to Figure 7. (A) Alignment of the LTR U3 DNA sequnces of tested SIVsmm (1-6) and HIV-2 variants (7-12) with the SIVmac239 sequence. Non-conserved positions are highlighted in yellow. (B) Stem-loop structure in the SIVmac239 RNA based on SHAPE RNA structure probing (67). Base-paired nucleotides are boxed and labelled (1L-1R, 2L-2R, 3L-3R) in panels A and B. (C) RNA fold structure analysis predicts a similar 1L-1R hairpin for SIVsmm L1 but not for HIV-2 7312A (but no 2L-2R or 3L-3R stem). CpG is indicated by a blue circle. Loop sequence indicated by red circle. (D*) Sequence variation in the 1L-1R hairpin region in tested SIVsmm (left) and HIV-2 strains (right). The SIVmac239 RNA hairpin structure is shown with the nucleotides differing in the SIVsmm and HIV-2 strains indicated. The numbers in superscript refer to the labelled sequences shown in panel D. Nucleotides in green allow basepairing, whereas nucleotides in red do not.*

Figure S8. *Related to Figure 4. (A) Sliding window (10bp) analysis of HIV-2 7312A and SIVsmmL1 nucleotide sequence identity based on aligned U3 sequences. Colours refer to U3 region A (green), B (blue) and C (pink). (B) Infectious virus yield of the WT and U3 mutant viruses in transfected HEK293T ZAP KO cells. N=3 + SD. **, P < 0.01; calculated using Student's t-test.*

Figure S9. *Related to Figure 8. Alignment of HA-ZAP eCLIP data to the 3'LTR of the HIV-2 7312A and SIVsmm genomic RNA. Relative coverage information in IP vs. size-matched input (SMI) is calculated at each position and displayed along the viral genome. Blue track – reads mapping to the positive strand; pink – reads mapping to the negative strand.*
